# Conformational proofreading of distant 40S ribosomal subunit maturation events by a long-range communication mechanism

**DOI:** 10.1038/s41467-019-10678-z

**Published:** 2019-06-21

**Authors:** Valentin Mitterer, Ramtin Shayan, Sébastien Ferreira-Cerca, Guillaume Murat, Tanja Enne, Dana Rinaldi, Sarah Weigl, Hajrija Omanic, Pierre-Emmanuel Gleizes, Dieter Kressler, Celia Plisson-Chastang, Brigitte Pertschy

**Affiliations:** 1 Institute for Molecular Biosciences, University of Graz, Humboldtstrasse 50, 8010 Graz, Austria; 2 Laboratoire de Biologie Moléculaire Eucaryote, Centre de Biologie Intégrative, Université de Toulouse, CNRS, UPS, 118 route de Narbonne, 31062 Toulouse Cedex, France; 3 Biochemistry III – Institute for Biochemistry, Genetics and Microbiology, University of Regensburg, Universitätsstraße 31, 93053 Regensburg, Germany; 4 Unit of Biochemistry, Department of Biology, University of Fribourg, Chemin du Musée 10, 1700 Fribourg, Switzerland; 5 Present Address: Biochemistry Centre, University of Heidelberg, 69120 Heidelberg, Germany

**Keywords:** RNA, Cell biology, Cryoelectron microscopy

## Abstract

Eukaryotic ribosomes are synthesized in a hierarchical process driven by a plethora of assembly factors, but how maturation events at physically distant sites on pre-ribosomes are coordinated is poorly understood. Using functional analyses and cryo-EM, we show that ribosomal protein Rps20 orchestrates communication between two multi-step maturation events across the pre-40S subunit. Our study reveals that during pre-40S maturation, formation of essential contacts between Rps20 and Rps3 permits assembly factor Ltv1 to recruit the Hrr25 kinase, thereby promoting Ltv1 phosphorylation. In parallel, a deeply buried Rps20 loop reaches to the opposite pre-40S side, where it stimulates Rio2 ATPase activity. Both cascades converge to the final maturation steps releasing Rio2 and phosphorylated Ltv1. We propose that conformational proofreading exerted via Rps20 constitutes a checkpoint permitting assembly factor release and progression of pre-40S maturation only after completion of all earlier maturation steps.

## Abstract (web summary)

The biogenesis of eukaryotic ribosomes is a multi-step process involving the action of more than 200 different ribosome assembly factors. Here the authors show that Rps20 acts as a conduit to coordinate maturation steps across the head domain of the nascent small ribosomal subunit.

## Introduction

Eukaryotic ribosomes consist of a large 60S and a small 40S subunit, each composed of ribosomal RNA (rRNA) and ribosomal proteins (r-proteins). The synthesis of ribosomes is a highly complex process starting with the assembly of pre-rRNA, r-proteins, and ribosome assembly factors (AFs) into pre-ribosomal particles in the nucleolus (reviewed in refs. ^1–4^). In yeast, the first steps in the synthesis of 40S subunits occur within large precursors termed SSU processomes or 90S particles, in which rRNA folding and processing steps, as well as incorporation of r-proteins and AFs, take place^5–9^. Endonucleolytic cleavage of the pre-rRNA, together with the dissociation of a large number of AFs, then results in the release of a 43S particle, also termed pre-40S particle, which contains a 3′ extended precursor of the mature 18S rRNA (the 20S pre-rRNA), most 40S r-proteins, and only a few AFs^2,4,10^. These particles are exported into the cytoplasm, where further 40S maturation events take place. Finally, conversion of the 20S pre-rRNA into the 18S rRNA by the endonuclease Nob1 results in the release of mature, translation-competent 40S subunits^11–13^.

Several biochemical studies together with recent cryo-electron microscopy (cryo-EM) structural analyses provided insights into the organization of early cytoplasmic pre-40S particles, thus revealing that AFs Tsr1, Pno1, Nob1, Dim1, and Rio2 are located on the intersubunit side of the pre-40S subunit and that Enp1 and Ltv1 are bound on the solvent-exposed side in the area of the beak structure^10,14–22^. These early cytoplasmic pre-40S particles undergo a cascade of maturation events, with the two first and presumably rate limiting ones being two different ATP-dependent maturation steps, resulting in dissociation of AFs Rio2 and Ltv1^14,19,23–26^.

Rio2 is an ATPase that is bound at the junction between the head and the body of pre-40S subunits and its release from pre-40S particles is promoted by ATP hydrolysis^14,27^. In addition, Rio2 release was suggested to require and trigger conformational changes necessary for progression of pre-40S particles into subsequent maturation steps^14,27^.

Ltv1 interacts with two 18S rRNA helices (h16 and h41) and several proteins (i.e., Enp1 and r-proteins Rps3 (uS3 according to the new nomenclature of r-proteins^28^) and Rps20 (uS10)) within the head domain of pre-40S particles, and its release involves at least two different steps. One is promoted by phosphorylation of three serines in the C-terminal part of Ltv1 by the casein kinase I homolog Hrr25^19,23,24^. The other step comprises the formation of contacts of the Rps3 N-domain with Rps20 and rRNA helix h41^19,29^. It is, however, not known whether these two steps are interconnected and in which order they occur.

Notably, mutations blocking Ltv1 release also inhibited the release of other AFs from pre-40S particles, including Rio2, indicating that Ltv1 dissociation is a prerequisite for the release of these factors^19^. However, previous studies on Rio2 demonstrated that a catalytically inactive *rio2* mutant, which is impaired in Rio2 release, accumulates Ltv1 on pre-40S particles, indicating that Rio2 catalytic activity and/or its subsequent release is, on the other hand, a prerequisite for Ltv1 release^14,30^. How Ltv1 release, taking place on the solvent side of the pre-40S subunit, might be coordinated with Rio2 catalytic activity and/or release, which occurs >50 Å apart on the intersubunit side (distance between the h41 Ltv1 rRNA-binding site, which is later occupied by the Rps3 N-domain, and the h31 Rio2 rRNA-binding site^15^), has however remained elusive.

Here we report that Rps20 coordinates Ltv1 and Rio2 release. The largest part of Rps20 is located on the solvent-exposed side of pre-40S particles where it contacts Rps3, thereby promoting Hrr25 recruitment by Ltv1 and subsequent Ltv1 phosphorylation. Two β-strands in Rps20 connected by an unstructured loop dive deeply into the (pre-)40S subunit, almost reaching to the Rio2-binding site. Deletion of this Rps20 loop leads to a reduction of Rio2’s ATPase activity; however, Ltv1 phosphorylation can still occur normally. Vice versa, *rps20* mutations preventing Ltv1 phosphorylation still allow Rio2 ATP hydrolysis. In either case, however, final release of both Rio2 and Ltv1 from pre-40S particles is inhibited. We conclude that Rio2 and Ltv1 release are multi-step processes, with the respective final steps occurring in an interdependent manner. Notably, pre-40S particles from an *rps20*Δloop mutant differ in several structural features from recently published wild-type pre-40S particles. We suggest that sensing of the correct conformations of both maturation sites, exerted by Rps20, provides a quality control checkpoint, which ensures that release of Ltv1 and Rio2 is only triggered once all necessary earlier maturation steps have been completed.

## Results

### Rps3 N-domain assembly promotes Hrr25 recruitment

Our previous studies suggested that both the phosphorylation of Ltv1 by Hrr25 and the contact formation between the Rps3 N-domain and Rps20 are required for Ltv1 release, but the order of these maturation events was unclear^19^. To better define the effects of mutations impairing the establishment of contacts between positively charged amino acids of the Rps3 N-domain (K7 and K10) and negatively charged amino acids of Rps20 (D113 and E115), we analyzed the composition of pre-40S particles isolated from such mutants. We considered the AF Tsr1 as a suitable bait protein for this purification, since it purifies a broad range of pre-40S particles by binding in the nucleus and dissociating at a late cytoplasmic step, after Ltv1 and Rio2 release^10,31,32^.

**Fig. 1:**
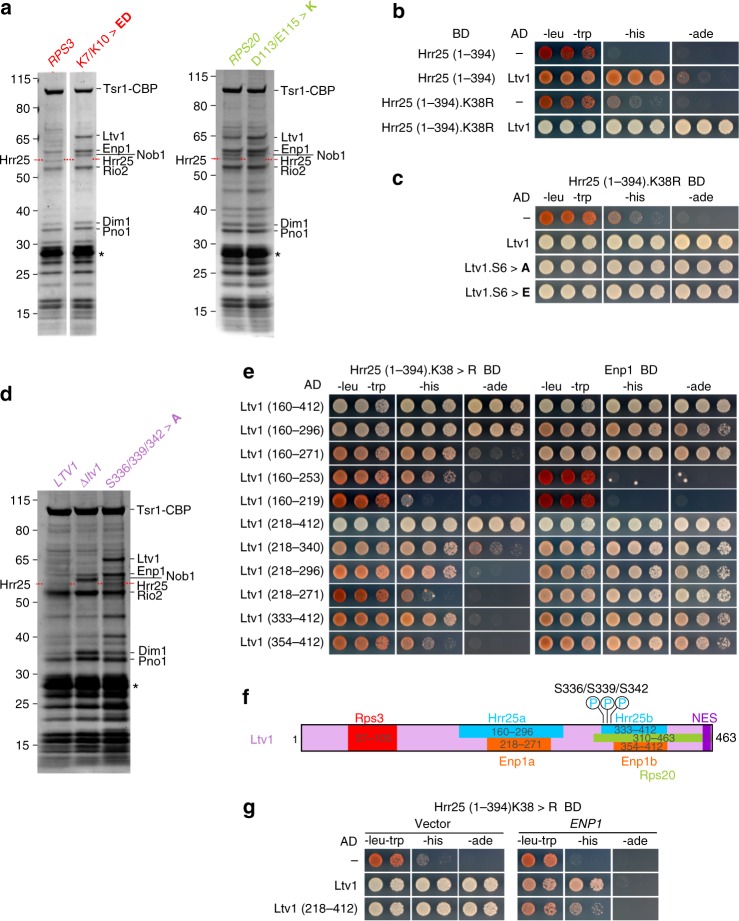
Rps3 N-domain assembly promotes Hrr25 recruitment. **a** Reduced binding of Hrr25 to particles impaired in Rps3 N-domain/Rps20 contact formation. Tsr1-TAP particles isolated from wild-type cells or from cells expressing the indicated *rps3* or *rps20* mutant alleles were analyzed by sodium dodecyl sulfate-polyacrylamide gel electrophoresis (SDS-PAGE) and Coomassie staining. Mass spectrometric (MS) analyses revealed that, while Hrr25 is present on particles isolated from wild-type strains, it is absent or strongly reduced from particles on which the Rps3 N-domain is not assembled (i.e., *rps3*.K7/K10>**ED** and *rps20*.D113/E115>**K**). Asterisks indicate the band of the Tobacco etch virus (TEV) protease used to elute pre-40S particles from the IgG beads. **b**, **c** Characterization of the interaction between Ltv1 and Hrr25. A C-terminal Hrr25 truncation (Hrr25(1–394)) and a mutant version thereof (Hrr25(1–394).K38R) fused to the Gal4 DNA-binding domain (BD) was tested for Y2H interaction with Ltv1 (**b**) and Ltv1 phosphomutants (Ltv1.S6>**A** and Ltv1.S6>**E**) (**c**) fused to the Gal4 activation domain (AD). Cells were spotted in ten-fold serial dilutions on SDC-Leu-Trp, SDC-His-Leu-Trp (-his; growth on this medium indicates a weak interaction), and SDC-Ade-Leu-Trp (-ade; growth on this medium indicates a strong interaction) plates. **d** Ltv1 is required to recruit Hrr25. Tsr1-TAP particles were isolated from *ltv1*Δ cells expressing plasmid-borne wild-type *LTV1*, the *ltv1*(S336/S339/S342>**A**) phosphomutant, or harboring an empty plasmid (Δ*ltv1*). Eluates were analyzed by SDS-PAGE, Coomassie staining, and MS. The asterisk indicates the TEV protease. **e** Hrr25(1–394).K38R (left panel) and Enp1 (right panel) fused to the BD were tested for Y2H interaction with the indicated Ltv1 fragments fused to the AD. **f** The deduced, minimal binding sites for Hrr25 and Enp1 on Ltv1 are indicated. In addition, the sites for Rps3 binding, Rps20 binding, and phosphorylation by Hrr25^19^, as well as the nuclear export sequence^35^, are depicted. **g** Enp1 overexpression weakens the Hrr25-Ltv1 interaction. Y2H strains expressing Hrr25(1–394).K38R fused to the BD in combination with the indicated Ltv1 constructs fused to the AD were transformed with empty plasmid (left panel) or with a *URA3* plasmid for *ENP1* overexpression under control of the *ADH1* promoter (right panel). Cells were spotted in ten-fold serial dilutions on SDC-Leu-Trp-Ura (panel: -leu-trp), SDC-His-Leu-Trp-Ura (panel: -his), and SDC-Ade-Leu-Trp-Ura (panel: -ade) plates

In line with our previous results, several AFs, including Ltv1, accumulated on pre-40S particles isolated from *rps3*.K7/K10>**ED** or *rps20*.D113/E115>**K** mutants (Fig. 1a). Surprisingly however, one band (indicated by a red dashed line in Fig. 1a) was significantly reduced in *rps3*.K7/K10>**ED** and *rps20*.D113/E115>**K** mutants. Analysis by mass spectrometry revealed that this band corresponded to the Hrr25 kinase, which executes phosphorylation of Ltv1^19,23,24^. Hence, formation of the Rps3-Rps20 contact occurs prior to and is even a pre-requisite for Ltv1 phosphorylation.

We speculated that restructuring of the Rps3 N-domain^19^ promotes recruitment of Hrr25 by unleashing a crucial binding surface. To better understand how Hrr25 is recruited to pre-40S particles, we performed yeast two-hybrid (Y2H) analyses testing the interaction of Hrr25 with several other AFs contained in pre-40S particles, as well as with some r-proteins of the 40S head domain (Supplementary Fig. 1a). Ltv1 showed a robust interaction with Hrr25, while no other tested AF interacted with Hrr25 in this assay and only the r-protein Rps15 displayed a weak interaction with Hrr25 (Supplementary Fig. 1a). Since full-length Hrr25 (494 aa) fused to the Gal4 DNA-binding domain displayed some self-activation of the *HIS3* reporter gene (Supplementary Fig. 1a, left panel), we performed subsequent analyses with a C-terminally truncated version of Hrr25 (aa 1–394)^33^, which showed no self-activation and still interacted with Ltv1, albeit with reduced efficiency (Fig. 1b). Moreover, mutation of the catalytic lysine residue to arginine (K38R mutant) strengthened the interaction between Ltv1 and this truncated (1–394) Hrr25 variant (Fig. 1b and Supplementary Fig. 1a, right panel). The interaction was fully maintained when the three main Hrr25 phosphorylation sites on Ltv1 (S336, S339, and S342) and an additional three proximal serines (S344, S345, and S346) were either exchanged for alanines (Ltv1.S6>**A**) or glutamates (Ltv1.S6>**E**), mimicking the unphosphorylated or phosphorylated states of these residues, respectively (Fig. 1c). Accordingly, Hrr25 was present in pre-40S purifications from the *ltv1*.S336/339/342>**A** mutant but absent in pre-40S preparations from a Δ*ltv1* strain (Fig. 1d). Together, these results suggest that Ltv1 is necessary to recruit Hrr25 to pre-40S particles and that Ltv1’s phosphorylated serines are only transiently bound by Hrr25 during its catalytic activity, while the main Hrr25 interaction site lies elsewhere within Ltv1.

The fact that Hrr25 is less efficiently recruited when the contact between the Rps3 N-domain and Rps20 is abolished suggests that a structural re-arrangement brings Ltv1 into a conformation where it is able to interact with Hrr25. To better understand which part of Ltv1 needs to be exposed to allow for Hrr25 recruitment, we mapped the Hrr25-binding sites on Ltv1. Remarkably, Ltv1 appears to harbor two sites, which can bind Hrr25 independently, and whose minimal fragments either exhibit a robust (aa 160–296) or moderate (aa 333–412) Y2H interaction with Hrr25(1–394).K38R (Fig. 1e and Supplementary Fig. 2). Notably, the second, weaker interaction surface comprises the residues that are phosphorylated by Hrr25 and partially overlaps with the previously mapped Rps20-binding region (Fig. 1f, ref. ^19^). Enp1 is another pre-40S component that directly interacts with Ltv1 via its bystin domain (aa 155–470^9^, Supplementary Fig. 1b) and was suggested to be a phosphorylation substrate of Hrr25^14,24,34^. Strikingly, Ltv1 also contains two independent Enp1-binding sites; both of these minimal Enp1-binding fragments (aa 218–271 and aa 354–412 of Ltv1) display an equally strong Y2H interaction and largely overlap with the Hrr25-binding regions (Fig. 1e, f and Supplementary Fig. 2). In line with this, overexpression of Enp1 weakened the Y2H interaction between Ltv1 and Hrr25, indicating that Enp1 and Hrr25 compete for Ltv1 binding (Fig. 1g). Therefore, we propose that the binding of Hrr25 helps to physically detach the interaction between Ltv1 and Enp1, thereby loosening the interaction of Ltv1 with pre-40S particles even before its phosphorylation and final dissociation take place.

### Cytoplasmic pre-40S maturation steps are functionally linked

Mutations blocking Ltv1 release also inhibited release of other AFs from pre-40S particles, including Rio2, while mutations impairing Rio2 release also accumulated Ltv1 on pre-40S particles, indicating that Ltv1 and Rio2 release are mutually interdependent^14,19,30^. To confirm that ATP hydrolysis by Rio2 is necessary for Ltv1 release, we made use of an Ltv1-GFP reporter construct that shows a nuclear steady-state localization due to mutation of Ltv1’s nuclear export sequence (NES) (Ltv1-NES3>**A**-GFP) (Supplementary Fig. 3a, refs. ^19,35^). As we showed previously, cytoplasmic Ltv1 release defects can be readily identified by means of a cytoplasmic mislocalization of such a reporter construct^19^. The localization of the reporter construct was evaluated in a mutant expressing the catalytically inactive *rio2*.D253A allele, which is viable but shows reduced Rio2 disassembly from 40S maturation intermediates^14^. Indeed, in contrast to its predominantly nuclear localization in wild-type cells, the Ltv1-NES3>**A**-GFP reporter construct mislocalized to the cytoplasm in *rio2*.D253A cells (Supplementary Fig. 3b). In addition, the Enp1-GFP fusion protein also accumulated in the cytoplasm in the *rio2*.D253A mutant, indicating a block of downstream pre-40S maturation steps.

**Fig. 2:**
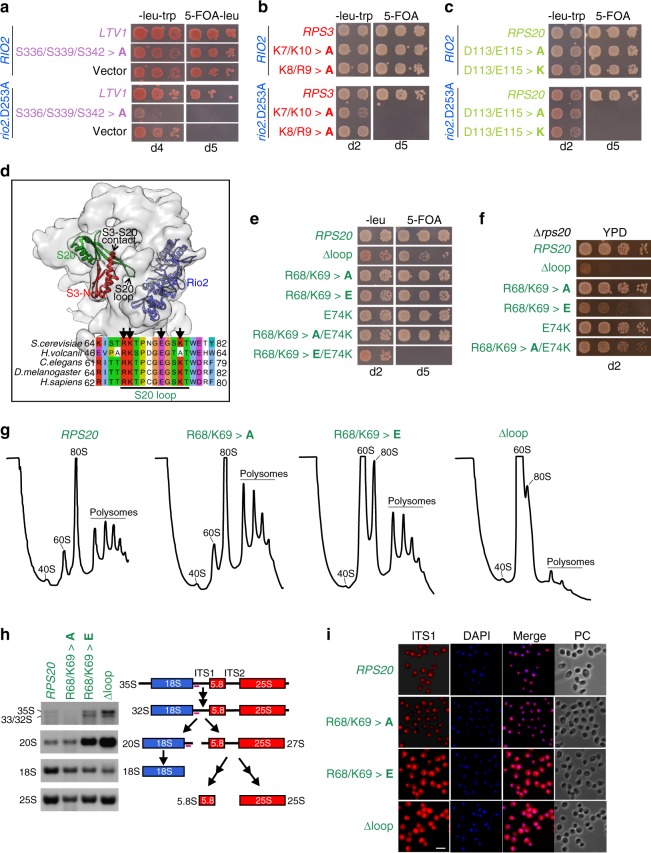
A conserved loop in Rps20 promotes cytoplasmic 40S subunit maturation. **a**–**c** Crucial cytoplasmic pre-40S maturation steps are functionally interconnected. A *RIO2* (*rio2*Δ) shuffle *ltv1*Δ strain (**a**), a *RIO2* (*rio2*Δ) *RPS3* (*rps3*Δ) double shuffle strain (**b**), and a *RIO2* (*rio2*Δ) *RPS20* (*rps20*Δ) double shuffle strain (**c**) were transformed with plasmids carrying the indicated wild-type and mutant alleles. Transformants were spotted in ten-fold serial dilutions on SDC-Leu-Trp (-leu-trp) or 5-FOA containing medium (to select for cells that have lost the respective *URA3* plasmid(s) harboring the wild-type gene(s)) and growth was monitored after incubation at 30 °C for the indicated days. **d** Rps20 loop reaches through the pre-40S subunit toward Rio2 on the intersubunit side. Rps20 (green), Rps3 N-domain (red), and Rio2 (blue) in the pre-40S structure (PDB 6FAI)^20^ (upper panel). Sequence alignment reveals conservation of the Rps20 loop between eukaryotes and archaea (lower panel). Arrows point to residues R68, K69, E74, and K77, which were mutated in this study. **e**, **f** Rps20 loop is crucial for cell growth. An *RPS20* (*rps20*Δ) shuffle strain was transformed with the indicated plasmid-based alleles and transformants were spotted in ten-fold serial dilutions on SDC-Leu and SDC+5-FOA plates (**e**) or, after plasmid shuffling on 5-FOA, on YPD plates (**f**). Growth was monitored after incubation at 30 °C for the indicated days. **g**–**i** Rps20 loop mutants strongly impair 40S subunit synthesis. **g** Polysome profiles of the indicated *rps20* loop mutants were recorded after centrifugation on 7–45% sucrose gradients. **h** Northern blot analyses of total RNA extracts from wild-type *RPS20* or the indicated *rps20* mutant cells using probes detecting the 20S pre-rRNA, mature 18S rRNA, and 25S rRNA (left panel). Simplified pre-rRNA processing scheme (right panel). All detected (pre-)rRNA species are indicated in bold letters. The binding site of the D/A_2_ probe used to detect the 20S pre-rRNA and precursors thereof is indicated in magenta. **i** Cells expressing wild-type *RPS20* or the indicated *rps20* alleles were analyzed by fluorescence in situ hybridization (FISH) using a Cy3-labeled probe specific to the D/A2 segment of internal transcribed spacer 1 (ITS1; depicted in **h**). The nucleoplasm was stained with DAPI. PC phase contrast. Scale bar is 5 µm

The interdependence of the spatially separated Rio2 and Ltv1 release events prompted us to test for functional connections between mutations affecting Rio2 release and mutations inhibiting Ltv1 release. To this end, we tested the *ltv1*.S336/S339/S342>**A** phosphorylation site mutant for genetic interaction with the catalytically inactive *rio2*.D253A variant, notably revealing a synthetic lethal phenotype (Fig. 2a). In addition, combining the *rio2*.D253A allele with *rps3* or *rps20* variants, which are impaired in establishing contacts between Rps3 (K7 and K10) and Rps20 (D113 and E115) or between Rps3 (K8 and R9) and rRNA (h41), resulted in synthetic lethal phenotypes (Fig. 2b, c). These results suggest a tight functional connection between Rps3 N-domain assembly, Ltv1 phosphorylation and release, and ATP-hydrolysis-dependent release of Rio2.

### A conserved Rps20 loop is essential for pre-40S maturation

While Ltv1, Rps3, and Rps20 are positioned on the solvent-exposed side of the pre-40S head domain, Rio2 is positioned on the subunit-interface side, bridging the head and the body^14–18,20–22^. We speculated that one or several proteins might physically link these distant sites to allow communication between them. We recognized that two long β-strands of Rps20 protrude deeply into the interior of the mature 40S subunit and an unstructured loop connecting these β-strands reaches almost to the opposite subunit-interface side^29^. Remarkably, this loop is in close contact with 18S rRNA helix h31, which also contains one of the rRNA-binding sites (nucleotides 1194–1196) of Rio2 in pre-40S particles, suggesting a cross-talk between the Rps20 loop and Rio2 either by a direct interaction or via their rRNA contact with helix h31 (Fig. 2d, refs. ^15,20^). The 11-amino-acid-long Rps20 loop (aa 68–78) contains four charged amino acids (R68, K69, K77, and E74), which might establish interactions with rRNA and/or proteins, and is highly conserved among eukaryotes (Fig. 2d).

To address the possibility that the Rps20 loop-region participates in 40S biogenesis, we analyzed the phenotypes resulting from deletion of the loop or from point mutations of the charged residues. Simultaneous substitution of amino acids R68, K69, and E74 to opposite charges (*rps20*.R68/K69>**E**/E74K) was lethal, whereas all other tested mutants were viable (Fig. 2e, f, Supplementary Fig. 3c, d). However, two of the combined point mutants (*rps20*.R68/K69>**E** and *rps20*.R68/K69>**A**/E74K), as well as deletion of the Rps20 loop (Δ68–78), caused a pronounced slow-growth phenotype (Fig. 2f, Supplementary Fig. 3c, d). Moreover, in contrast to wild-type cells and the *rps20*.R68/K69>**A** mutant, the *rps20*.R68/K69>**E** and, to an even greater extent, the *rps20*Δloop mutant displayed a 40S synthesis defect in polysome analyses, as evidenced by a strong increase of free 60S subunits (Fig. 2g). Moreover, presumably as a consequence of the severe imbalance between 40S and 60S subunits, the *rps20*Δloop mutant also showed significantly reduced polysome levels. Northern blot analyses revealed a reduction of the mature 18S rRNA and a substantial accumulation of its direct precursor, the 20S pre-rRNA, in the *rps20*.R68/K69>**E** and the *rps20*Δloop mutant (Fig. 2h). Furthermore, fluorescence in situ hybridization showed that the 20S pre-rRNA accumulated in the cytoplasm, demonstrating that the inhibition of the pre-40S maturation pathway in *rps20* loop mutants occurs at the stage of cytoplasmic pre-40S particles (Fig. 2i). Since cytoplasmic 20S pre-rRNA accumulation was observed as well upon depletion of Rps20^36^, we wanted to exclude the possibility that the observed phenotypes are the consequence of a failure to incorporate Rps20, mimicking depletion of the protein. To this end, we assessed the levels of N-terminally HA-tagged Rps20 variants in purified pre-40S particles by western blotting with anti-HA antibodies. Notably, not only the Rps20Δloop and Rps20.R68/K69>**E** but also the Rps20.D113/E115>**K** variant were fully incorporated into pre-40S particles, indicating that the observed phenotypes are specific maturation defects caused by the structural alterations introduced into the Rps20 protein (Supplementary Fig. 4).

### The Rps20 loop is genetically linked to 40S maturation

**Fig. 3:**
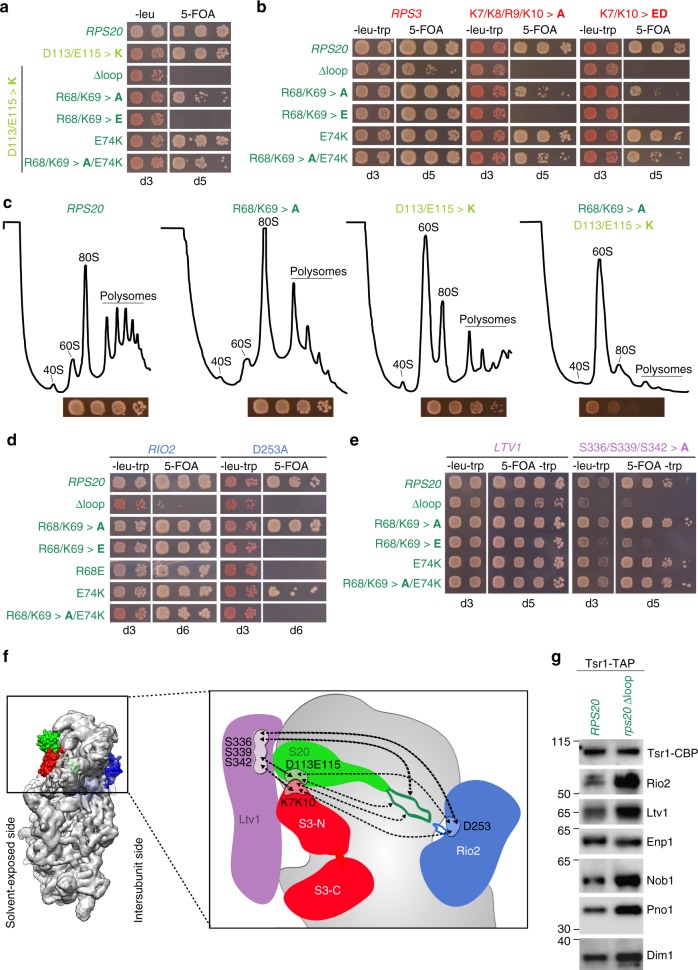
A genetic network between Rps20 loop and 40S maturation mutants. **a**–**c** *rps20* loop alleles enhance the phenotypes of mutants that impair Rps3 N-domain assembly. **a** An *RPS20* (*rps20*Δ) shuffle strain was transformed with the indicated plasmid-based *rps20* alleles. Transformants were spotted in ten-fold serial dilutions on SDC-Leu and SDC+5-FOA plates and growth at 30 °C was monitored after 3 and 5 days, respectively. **b** An *RPS20* (*rps20*Δ) *RPS3* (*rps3*Δ) double shuffle strain was transformed with the indicated plasmid-based *RPS20* and *RPS3* wild-type and mutant alleles. Transformants were spotted in ten-fold serial dilutions on SDC-Leu-Trp and SDC+5-FOA plates and growth at 30 °C was monitored after 3 and 5 days, respectively. **c** Polysome profiles of cells expressing the indicated *rps20* alleles, revealing an increased 40S synthesis defect when a *rps20* loop mutation is combined with a *rps20* allele that affects the interaction with the Rps3 N-domain (R68/K69>**A** D113/E115>**K** mutant). The panels below the profiles show growth of the respective strains on YPD plates incubated for 2 days at 30 °C. **d**, **e** Genetic interactions between *rps20* loop alleles and mutants of pre-40S assembly factors (AFs). An *RPS20* (*rps20*Δ) *RIO2* (*rio2*Δ) double shuffle strain (**d**) and an *RPS20* (*rps20*Δ) shuffle *ltv1*Δ strain (**e**) were transformed with the indicated plasmid-based alleles. Transformants were spotted in ten-fold serial dilutions on SDC-Leu-Trp (-leu-trp) or 5-FOA-containing medium and growth at 30 °C was monitored at the indicated days. **f** Genetic network illustrating the interplay of the Rps20 loop with AFs and r-proteins at distant sites on the pre-40S ribosome. All genetic interactions discovered in this study (Figs. 2 and 3) and in our previous study^19^ are indicated by dashed lines. **g** The Rps20 loop is required for AF release from cytoplasmic pre-40S particles. Tsr1-TAP particles were purified from wild-type *RPS20* or *rps20*Δloop yeast strains. Eluates were analyzed by sodium dodecyl sulfate-polyacrylamide gel electrophoresis and western blotting using the indicated specific antibodies

To get insights into whether the Rps20 loop could participate in the crucial steps leading to Ltv1 and Rio2 release, we undertook genetic analyses. Intragenic combination of the *rps20*Δloop deletion or the *rps20*.R68/K69>**E** mutation with the *rps20*.D113/E115>**K** mutation, which prevents binding of Rps20 to the Rps3 N-domain^19^, abolished cell growth (Fig. 3a). In line with this, we also observed synthetically enhanced growth phenotypes when combining the *rps20* loop-mutants with mutants in which the Rps3-Rps20 contact was reduced from the Rps3 side (Fig. 3b). Moreover, polysome analyses revealed that the 40S synthesis defect of the *rps20*.D113/E115>**K** mutant is strongly enhanced by the R68/K69>**A** loop-mutation (Fig. 3c). Remarkably, we also found synthetic lethal phenotypes of *rps20* loop-mutants, including mutants that display no growth phenotype on their own, with the catalytically inactive *rio2*.D253A mutant (Fig. 3d). Furthermore, *rps20* loop-mutants severely enhanced the growth defect of the *ltv1*.S336/S339/S342>**A** phosphomutant (Fig. 3e). Taken together, we unraveled a genetic network (schematically depicted in Fig. 3f) exhibiting multifaceted interconnections between AFs and r-proteins and linking the Rps20 loop to cytoplasmic pre-40S restructuring events and Rio2 ATPase activity.

### The Rps20 loop is required for release of AFs

To address whether the Rps20 loop participates in release of Rio2 and/or Ltv1, we purified pre-40S particles from *rps20*Δloop cells using again Tsr1-TAP as bait protein. These analyses revealed that Rio2 strongly accumulated on pre-40S particles isolated from the *rps20*Δloop mutant (Fig. 3g, Supplementary Fig. 5). In addition, the levels of Ltv1 and of downstream maturation factors like Nob1 and Pno1 increased, suggesting that the loop deletion blocks pre-40S maturation at an early cytoplasmic step prior to Ltv1 and Rio2 disassembly. The strong accumulation of Rio2 further strengthens the hypothesis that the Rps20 loop-region is directly involved in release of the Rio2 ATPase.

### Rio2 and the Rps20 loop trigger final release of Ltv1

**Fig. 4:**
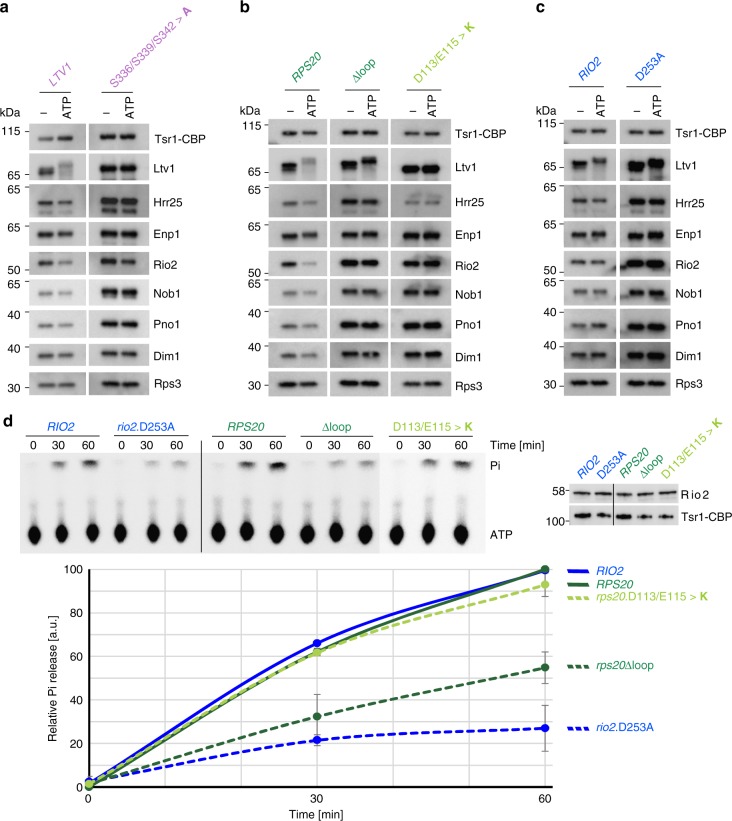
Rio2 ATP hydrolysis stimulated by the Rps20 loop triggers release of Ltv1. **a**–**c** In vitro phosphorylated Ltv1 remains trapped on pre-40S particles containing the catalytically inactive Rio2.D253A or the mutant Rps20Δloop protein. Tsr1-TAP particles from cells expressing wild-type or mutant alleles of *LTV1* (left panel), *RPS20* (middle panel), and *RIO2* (right panel) were immobilized on IgG beads. Particles were incubated in the absence (−) or presence (ATP) of ATP, washed, and eluted with TEV protease. Eluates were analyzed by western blotting using the indicated antibodies. The displayed blots within each section (**a**–**c**) originate from the same membrane and the same exposures, allowing for direct comparison of the levels of detected proteins. **d** The Rps20 loop stimulates Rio2 ATPase activity. Relative ATPase activity obtained from purified Tsr1-TAP particles, carrying the indicated *RIO2* or *RPS20* alleles, was monitored by single-turnover experiment. The input was adjusted to equal amounts of Rio2 (right panel). An exemplary thin layer chromatography showing ATP and released phosphate (Pi) is depicted (upper left panel). Mean values from quantification of the results obtained from two biological and two technical replicates are plotted (lower panel). Error bars represent standard deviations. Source data are provided as a Source Data file

To study the influence of the different mutants on ATP-dependent Ltv1 phosphorylation and release of Ltv1 and Rio2, we performed in vitro ATP incubation assays with purified pre-40S subunits. Tsr1-TAP particles were immobilized on IgG-Sepharose beads, incubated in the presence or absence of ATP and, after a subsequent washing step, eluted with Tobacco etch virus (TEV) protease. Notably, we observed an Ltv1 double band already in the untreated wild-type particles, likely corresponding to partial phosphorylation of Ltv1 in a subpopulation of the preparation (Fig. 4a, *LTV1*; Fig. 4b, *RPS20*; and Fig. 4c, *RIO2*). After incubation of wild-type particles with ATP, we observed a significant band-shift of both Ltv1 bands due to phosphorylation by Hrr25 (Fig. 4a–c); however, the phosphorylated form of the protein was largely released from these particles. In addition, also the association of Rio2 with wild-type particles diminished upon ATP incubation.

As expected, Ltv1 was neither phosphorylated nor released upon ATP incubation of particles purified from a strain in which serines 336, 339, and 342 of Ltv1 were exchanged for alanines, preventing phosphorylation of Ltv1, despite the enrichment of Hrr25 in these particles (Fig. 4a). In contrast, Hrr25 levels were reduced in pre-40S particles from the *rps20*.D113/E115>**K** mutant, confirming that assembly of the Rps3 N-domain is important for Hrr25 recruitment (Fig. 4b). Notably, Ltv1 was not phosphorylated and remained fully attached to these particles, as well as to particles from the *rps3*.K7/K10>**ED** mutant (Fig. 4b and Supplementary Fig. 6). As Ltv1 was not phosphorylated at all, despite the presence of residual levels of Hrr25 in pre-40S particles from the *rps20*.D113/E115>**K** mutant, we speculate that the low amounts of Hrr25 recruited in these mutants may not be correctly positioned relative to Ltv1 to perform the phosphorylation. Moreover, also Rio2 release was inhibited in these particles (Fig. 4b).

ATP-dependent Rio2 release was also blocked in pre-40S particles derived from the *rps20*Δloop mutant (Fig. 4b), as well as the *rio2*.D253A mutant (Fig. 4c). Most remarkably, however, Ltv1 was fully phosphorylated but nevertheless remained associated with these pre-40S particles, together with Hrr25, thus clearly indicating that the Ltv1 release cascade proceeds in *rps20*Δloop and *rio2*.D253A mutants until Ltv1 phosphorylation, but final dissociation of Ltv1 and Hrr25 from pre-40S particles is inhibited.

Together, the results of this assay suggest the following cascade of events culminating in Ltv1 release: the Rps3 N-domain re-orients and forms its contact with Rps20, thereby providing the prerequisites for Ltv1 phosphorylation by Hrr25. Concomitant repositioning of the Rps20 loop and ATP hydrolysis by Rio2 finally promote dissociation of phosphorylated Ltv1 from pre-40S particles.

Since ATP-dependent Rio2 release was not only inhibited in pre-40S particles from the *rio2*.D253A mutant (Fig. 4c) but also in both tested *rps20* mutants (Fig. 4b), we wondered whether all of these mutations prevented ATP hydrolysis by Rio2 or whether other effects were blocking Rio2 release. To address this question, we analyzed Rio2 ATP hydrolysis in pre-40S particles purified from different mutants by single-turnover experiments (Fig. 4d). As expected, wild-type particles were able to hydrolyze ATP, while ATP hydrolysis was largely reduced in the catalytic *rio2*.D253A mutant, confirming that Rio2 is the main protein contributor in the purified pre-40S particles hydrolyzing ATP and releasing the resulting phosphate. Strikingly, ATP hydrolysis was substantially reduced in the *rps20*Δloop mutant, revealing that the Rps20 loop plays a crucial role in activating the Rio2 ATPase. In contrast, ATP hydrolysis was not reduced in particles from the *rps20*.D113/E115>**K** mutant, indicating that Rio2 can hydrolyze ATP but is afterwards kept from dissociating from pre-40S particles (see Fig. 4b).

In conclusion, our results indicate a strong cooperativity between Ltv1 and Rio2 release, with both events being multi-step processes: the initial steps (Rps3-Rps20 contact formation, phosphorylation of Ltv1, and ATP hydrolysis by Rio2) can occur independently of the events on the respective other side of the 40S head, while the final steps, resulting in the dissociation of both factors, depend on the successful execution of all earlier steps. Moreover, our data suggest that Rps20, by sensing the correct Rps3 N-domain positioning via its Rps3-interacting residues D113 and E115 as well as the correct Rio2 maturation stage via its loop region, coordinates these distantly occurring release events.

### Rps20Δloop pre-40S particles are trapped in distinct states

**Fig. 5:**
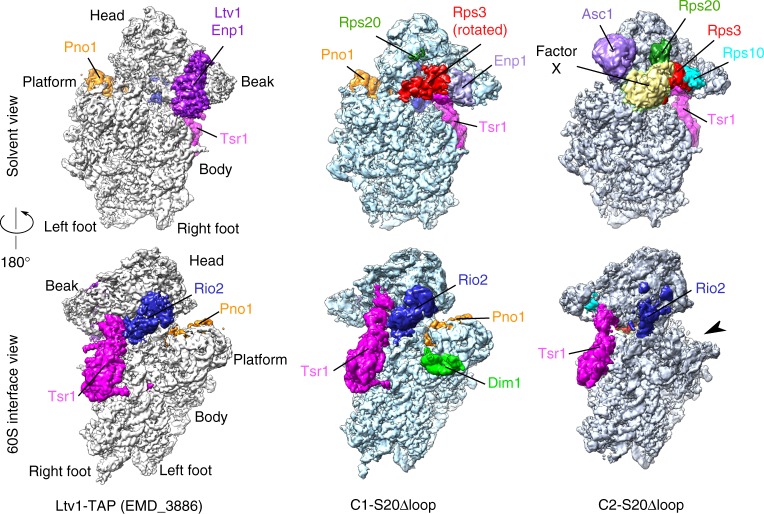
Cryo-electron microscopic (cryo-EM) analysis of Rps20Δloop pre-40S particles. Surface views of cryo-EM maps of Ltv1-purified wild-type pre-40S particles^16^ (left panel) and the Rps20Δloop pre-40S particles (central panel: C1-S20Δloop particles; right panel: C2-S20Δloop particles). Assembly factors and r-proteins of interest have been segmented and colored; the arrowhead next to the C2-S20Δloop map indicates missing density in the platform region compared to the other maps

We speculated that the delay in pre-40S maturation upon Rps20 loop deletion may also be reflected at the structural level and may trap particles in distinct, otherwise potentially short-lived, maturation stages. To determine three-dimensional (3D) structures of pre-40S particles from *rps20*Δloop mutant cells, purified via Tsr1 as bait, we used cryo-EM and single particle analysis. Initial processing allowed us to isolate a pool of particles showing a very stable and well-resolved body (consensus 3D structure in Supplementary Fig. 7a), whose features appeared similar to those of available pre-40S structures^16,20^. The head of the particles was much more blurred, which led us to perform focused 3D classifications on this region^37^. This resulted in separation of pre-40S particles into two distinct populations, hereafter called C1-S20Δloop and C2-S20Δloop representing 16% and 12%, respectively, of the 344,959 purified particles, which were initially included in the single particle analysis scheme (Fig. 5 and Supplementary Fig. 7a). The structures of the C1-S20Δloop and C2-S20Δloop particles could be solved at an average of 3.47 and 3.79 Å, respectively (Supplementary Fig. 7b–d). In both classes, the body is very well resolved (3.0–3.2 Å; Supplementary Fig. 7d), while, as previously observed in pre-40S particle structures^16,20,22^, the resolution of the head domain is lower (~3.6–6 Å in C1- and C2-“head only,” reconstructed with images where the signal of the body was subtracted; ~5–7 Å in C1-S20Δloop and C2-S20Δloop structures, arising from full particles; Supplementary Fig. 7b, d), hinting at higher structural dynamics in this region. The high resolution of these 3D reconstructions allowed us to build and refine an atomic model for both classes of particles, based on a published pre-40S structure for the C1-S20Δloop class^20^, and on the mature 40S subunit for the C2-S20Δloop class^29^ (Supplementary Table 1).

**Fig. 6:**
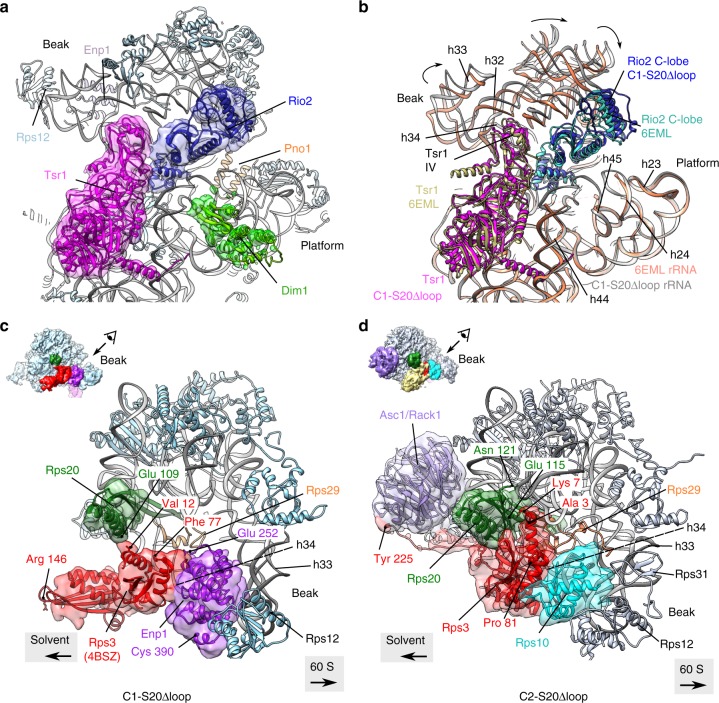
Structural details of C1-S20Δloop and C2-S20Δloop pre-40S particles. **a** Close-up of the atomic model of C1-S20Δloop, as seen from the 60S interface; segmented cryo-electron microscopic (cryo-EM) densities corresponding to Tsr1, Rio2, and Dim1 are shown in magenta, blue, and lime green, respectively. rRNA is in gray, and other r-proteins in pale blue. **b** Same view of the C1-S20Δloop model as in **a**, superimposed to the model of pre-40S particles purified with Ltv1 as bait (PDB 6EML)^16^. Spatial alignment was realized using the Matchmaker option in Chimera, using C1-S20Δloop rRNA as reference. For more clarity, only Tsr1, Rio2, and rRNA of both models are displayed. Color codes are indicated on the panel. Arrows indicate the rotation of the head of C1-S20Δloop particles compared to the PDB 6EML model. **c**, **d** Close-up of the heads of C1-S20Δloop (**c**) and C2-S20Δloop pre-40S particles (**d**), as seen from the top of the head/beak of the particles (top-left insets indicate direction of view). Segmented cryo-EM densities corresponding to Rps20, Rps3, Enp1, Asc1, and Rps10 are represented in green, red, purple, pale violet, and turquoise, respectively. rRNA is in gray, and other r-proteins in pale blue

The C1-S20Δloop class displays body and platform domains globally similar to those of other structures of yeast pre-40S particles (Fig. 5, compare C1-S20Δloop with the cryo-EM map of wild-type Ltv1-TAP purified particles (EMDB 3886^16^)). The main differences are within the head and at the bottom of the platform region. In the beak region, we were able to partly fit the solvent-exposed domain of Rps20 close to its mature position (Supplementary Fig. 10a, d); however, the cryo-EM maps harbored no clear density for the two β-strands that deeply dive into the pre-40S head. This suggests that Rps20 is not yet fully accommodated and stabilized in its mature position. The overall conformation of Rio2 in C1-S20Δloop particles was similar as in other pre-40S particles^16^ (Fig. 6a, b), with the C-lobe domain of Rio2 adopting an open conformation compared to the ATP-bound form of *Archaeoglobus fulgidus* Rio2^38^, likely preventing ATP hydrolysis by Rio2 (Supplementary Fig. 8b).

Notably, however, C1-S20Δloop pre-40S particles displayed several differences to published pre-40S structures on the solvent side of the head domain. We and others previously observed that, before its assembly into pre-40S particles, Rps3 is bound to its dedicated chaperone Yar1 in a conformation in which the C-domain is rotated relative to the N-domain (compared to 40S-bound Rps3)^19,39^. Strikingly, we observed a density detached from the head in the vicinity of rRNA helix h34, in which we could unambiguously rigid-body fit the X-ray structure of Rps3 in this rotated conformation (Figs. 5 and 6c and Supplementary Fig. 10a). While the Rps3 C-domain protrudes from the pre-40S structure, the Rps3 N-domain is bound to this pre-40S particle but in a different orientation than in mature 40S subunits (Supplementary Fig. 10a, d). As the residues in Rps3 (K7 and K10) and Rps20 (D113 and E115) interacting with each other in 40S subunits are not visible in this structure, it is unclear whether the contact between the Rps3 N-domain and Rps20 has already formed. We could not distinguish Ltv1 in the structure, suggesting that it is very flexible. Enp1 was also more flexible than in other structures, but we could nevertheless rigid-body fit its central part (aa 252–390, compared to aa 205–465 resolved in wild-type pre-40S particles^16^) next to rRNA helix h34 (Fig. 6c).

In the platform region, not only the 3′ end of mature 18S rRNA, which is protected by Pno1, but also the first two nucleotides of ITS1 are distinguishable in the C1-S20Δloop cryo-EM map (Supplementary Fig. 8c). Under the platform region on the 60S interface, C1-S20Δloop particles harbor protruding densities; focused classification of this region allowed us to unambiguously fit the alpha-helical C-terminal moiety of Dim1 (amino acids 136–318) into these densities (Figs. 5 and 6a and Supplementary Fig. 7a). Dim1 is absent from most previously reported yeast and human pre-40S structures^16,20,22^ and was only observed in a small sub-population of particles purified from a catalytically inactive Nob1 mutant^20^. We speculate that C1-S20Δloop particles are trapped at a stage in which Dim1 is in a more stable conformation. Last but not least, although Tsr1 is in a similar position as observed before, domain IV of the protein^40^ is slightly shifted toward the solvent side, hence pushing the head rRNA closer to its mature position (Fig. 6b).

The second structural class, C2-S20Δloop, resembles in many aspects mature 40S subunits (Supplementary Fig. 9). The head of C2-S20Δloop particles possesses densities clearly accommodating the full structures of Rps20, Rps3, and the presumably late assembling Rps10 and Asc1 (Figs. 5 and 6d, and Supplementary Fig. 10b), all in their mature position, whereas neither Ltv1 nor Enp1 are visible in the structure. As Rps10 occupies part of their binding sites, they have either already dissociated completely or, alternatively, have partly detached from the particles and are therefore too flexible to be visible.

Surprisingly, C2-S20Δloop particles harbor an additional large density protruding from the beak region in front of Rps3, with a position and a shape similar to that of the unidentified factor X in human late cytoplasmic pre-40S particles^22^ (Fig. 5 and Supplementary Fig. 9a, yellow density in C2-S20Δloop panels). Our attempts to gain resolution in this region by performing focused classifications were unsuccessful, suggesting that the pre-40S association of this factor X is highly labile. Given that Hrr25 is enriched in Rps20Δloop pre-40S particles (Fig. 4b), and factor X is moreover ideally placed on the head close to the binding region of Ltv1, Hrr25 is a good candidate for factor X. We thus used cryo-EM map segmentation to isolate the density from the rest of the EM map, followed by rigid-body docking of the X-ray structure of Hrr25 (PDB 5CZO)^33^ into this density. Indeed, the central domain of Hrr25 (aa 85–359) could be fitted, although only with a correlation coefficient of ~0.51 (Supplementary Fig. 11), preventing unambiguous assignment of Hrr25 to this density. Nevertheless, these results further support that Hrr25 is a possible candidate for this unidentified factor on our C2-S20Δloop particles.

On the intersubunit side of C2-S20Δloop particles, the upper part of rRNA helix h44 is still detached from the body as typical for pre-40S particles (Supplementary Fig. 9a, b). Moreover, the platform region is completely blurred (and thus averaged out), probably due to a wriggling movement of Rps1, Rps14, Pno1, and 18S rRNA helices h23 and h45 (right panel in Fig. 5, region indicated by an arrow, and Supplementary Fig. 9c). C2-S20Δloop particles also display fragmented densities where Rio2 and Tsr1 are located on C1-S20Δloop and previously described pre-40S particles (Fig. 5 and Supplementary Fig. 9a), suggesting that, although highly flexible, besides the bait protein also Rio2 is still associated with this otherwise more mature class of pre-40S particles.

To sum up, our cryo-EM analyses revealed that the maturation defects arising in the absence of the Rps20 loop trap pre-40S particles in distinct structural states displaying several differences to previously reported structures.

## Discussion

**Fig. 7:**
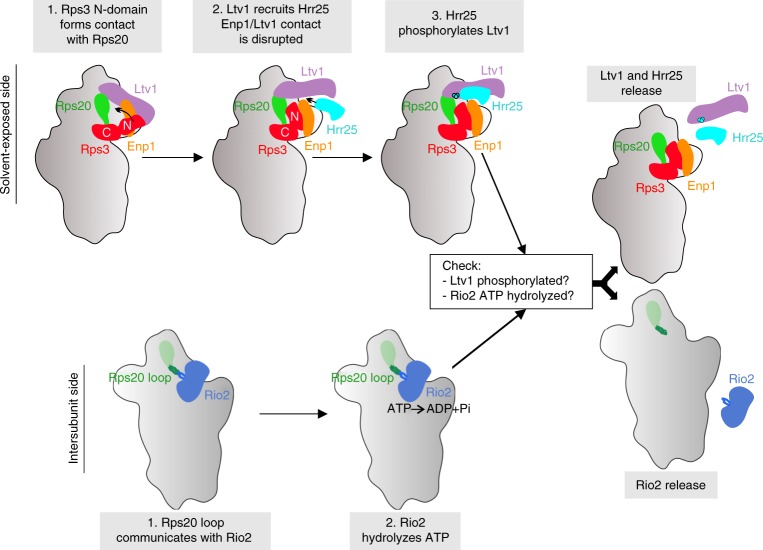
Model of Rps20-mediated coordination of cytoplasmic pre-40S maturation events. Maturation events on the solvent-exposed and intersubunit side of pre-40S subunits converge into a final maturation event leading to the release of Ltv1 and Rio2 (see “Discussion”)

In this work, we unraveled the intricate mechanism leading to the release of AFs Ltv1 and Rio2 from pre-40S particles, which is coordinated by the r-protein Rps20 (Fig. 7). On the solvent-exposed side of pre-40S particles, the Rps3 N-domain re-orients and forms its contact with Rps20. This conformational re-arrangement not only presumably weakens Ltv1’s interaction with pre-40S particles^19^ but also positions Ltv1 in a way that it can efficiently recruit Hrr25. Owing to competition for overlapping binding sites, Hrr25 likely (partially) disrupts the interaction between Ltv1 and Enp1. Hrr25 then phosphorylates Ltv1 and thereby further destabilizes its association. On the intersubunit side, the Rps20 loop stimulates ATP hydrolysis by Rio2. Our data show that, while mutants lacking the Rps20 loop are viable and cold-sensitive, as are catalytic Rio2 mutants^14^, the combination of both is lethal. This suggests that the Rps20 loop has, apart from its requirement for ATP hydrolysis, a second role, possibly by establishing the structural context needed for final Rio2 release. Most outstandingly, all the above described maturation events on the solvent-exposed and on the intersubunit side can take place independently of each other; however, the ultimate release of both Ltv1 and Rio2 is inhibited as soon as the maturation cascade is disrupted on either side. We propose that only the correct positioning of Rps20 on both sides commits the particles to proceed to Ltv1 and Rio2 dissociation. The eventual triggers for release of Rio2 and Ltv1 are not known. However, based on this study and recent data^27^, we speculate that Rps20 and/or rRNA re-arrangements are involved. The two AFs may either dissociate at the same time or one after the other. Indeed, there is evidence that Rio2 may dissociate after Ltv1^31,32^.

Comparison of recent cryo-EM structures representing different yeast and human pre-40S maturation intermediates^16,20,22^ with our C1- and C2-S20Δloop pre-40S structures (Figs. 5 and 6) can help to position our particles in a structural pre-40S maturation timeline.

Notably, some of the previously reported pre-40S structures^16,22^ lack densities for Rps3 and Rps20, despite biochemical evidence that they are already assembled at this maturation stage (see, for example, refs. ^19,21,41^). This suggests high flexibility of the region Rps3 and Rps20 are bound to. Both Rps3 and Rps20 are partly visible in our C1-S20Δloop particles, indicating that they are in a maturation stage where Rps3 and Rps20 have already been partly stabilized. Nevertheless, the Rps20 β-strands are not visible and the Rps3 C-domain sticks out of the pre-40S particles. Moreover, the Rps3 N-domain is in a different orientation than in mature 40S subunits, supporting our previous suggestion that the Rps3 N-domain is initially assembled in an incorrect orientation^19^.

The actual residues responsible for the interaction between the Rps3 N-domain and Rps20 are not resolved in our C1-S20Δloop structure; therefore, it remains subject to speculation whether this contact has already formed even though the Rps3 N-domain has not yet moved into its final position or whether the structural re-orientation of the Rps3 N-domain and contact formation with Rps20 are coupled and have not occurred yet in this population of S20Δloop particles. Importantly, however, the properties of pre-40S particles incapable of forming the Rps3-Rps20 contact clearly differ from Rps20Δloop particles (Fig. 4), for example, in the ability to recruit Hrr25. Interestingly, the pre-40S structure from the Ban laboratory^20^ shows a maturation stage where Rps20 and the Rps3 N-domain have already been accommodated in their mature position, while the Rps3 C-domain still sticks out of the particles, suggesting that stable integration of the Rps3 C-domain occurs even later than Rps3 N-domain accommodation. The step-wise incorporation of Rps3 is in line with previously published results showing that both Rps3 and Rps20 initially bind only loosely to pre-40S particles and their stable incorporation occurs at a later stage^24,41^.

Our cryo-EM structural analysis revealed no altered positioning of Rio2 in C1-S20Δloop particles. It is possible that the absence of the Rps20 loop leads to changes in flexible regions that are not resolved in the structure. Alternatively, the transition from the open conformation of Rio2 to the closed, ATP hydrolysis competent state, which is likely impeded in Rps20Δloop particles, might quickly trigger Rio2 ATP hydrolysis followed by its dissociation, a process that presumably is kinetically too fast to be captured by a static method like cryo-EM.

Our biochemical results strongly suggest that there is a communication between the Rps20 loop and Rio2. We suggest two possible scenarios how this could occur: (1) Both the Rps20 loop and Rio2 directly interact with 18S rRNA helix 31^20,29^; therefore, communication between the Rps20 loop and Rio2 may occur via h31. Notably, a recent study revealed that Rio2 ATPase activity is inhibited by RNA^27^. Hence, re-arrangements in, or dependent on the Rps20 loop may reposition inhibitory rRNA elements, thereby relieving the inhibition of Rio2. (2) An alternative possibility would be a direct interaction between the Rps20 loop and Rio2. Indeed, Rio2 also contains an unstructured flexible loop, which has not been resolved in any of the so far published X-ray or cryo-EM structures. However, the last two resolved amino acids, lysine 129 and serine 145, are positioned close enough to the Rps20 loop^20^ that such a contact would theoretically be possible. Since, however, neither the Rio2 flexible loop nor rRNA helix h31 are visible in our C1-S20Δloop structure due to flexibility, our structure does not provide further evidence for either model.

It is puzzling that, despite the massive effects of Rps20 loop deletion on pre-40S maturation, *rps20*Δloop cells are viable. This suggests that at least some pre-40S particles can mature into translation-competent 40S subunits. We hypothesize that the second structural class we observed, C2-S20Δloop, comprising ~12% of the particles in the purification, could represent particles that have escaped the blockade posed by the absence of the Rps20 loop. Alternatively, they might represent particles trapped in a dead end due to the inability to release the remaining AFs like Rio2 and Hrr25. In C2-S20Δloop particles, Rps3 and Rps20 are already in their mature position and Rps10 is assembled, while Ltv1 and Enp1 have either partly dissociated or completely left the particle. Moreover, Rio2 and Tsr1 seem to be only loosely attached to these particles. Interestingly, these particles contain a factor X, which was previously also observed in a late intermediate in a series of human pre-40S structures representing different maturation stages^22^. The factor might be Hrr25, which is enriched in Rps20Δloop pre-40S particles purified via Tsr1-TAP (Fig. 4b); the presence of Hrr25 in such late particles is, however, unexpected and needs to be verified in future studies, especially since Hrr25 appears to require Ltv1 for its efficient binding to pre-40S particles (Fig. 1), while it is unclear whether Ltv1 is still present in the C2-S20Δloop subpopulation.

Although cryo-EM analyses gave important insights into early cytoplasmic pre-40S maturation, it has to be mentioned that the picture obtained from these structural investigations remains incomplete. Indeed, no published structures cover the totality of all distinct particles present in the given bait purification, and often, to reach near-atomic resolutions, a majority of particles has to be omitted from 3D reconstructions because of their flexibility or ill-resolved features. In our case, as frequently in cryo-EM studies, our high-resolution maps included ~30% of the purified pre-40S particles. Furthermore, despite the great progress in the field gained in the last few months, cryo-EM analyses have only provided snapshots describing structurally stable states of purified macromolecules. None of the so far published pre-ribosomal structures were able to fully temporally or causally resolve interdependent, multi-step maturation cascades as we have revealed here by a combination of genetic, biochemical, and structural approaches. Thus our study exemplifies that only by combining structural knowledge with in-depth functional analyses it is possible to unravel individual steps of the pre-ribosomal maturation pathway at a mechanistic level.

Several hundred maturation factors participate in ribosome biogenesis, and it has to be an immense logistic challenge to coordinate their action. A consecutive order in which the point of action of each factor is precisely defined would be highly inefficient, as every single factor would need to communicate with many different, remote factors. Communication across the ribosomal subunit at certain checkpoints, as performed by Rps20 in the Rio2/Ltv1 release pathway, is a much more sophisticated way to coordinate the action of ribosome AFs. Interestingly, additional examples lead to the proposal that communication across nascent ribosomal subunits may play a role in coordinating ribosome biogenesis (see, for example, refs. ^27,42,43^); however, the molecular mechanisms underlying these processes remain subject to future investigations. Moreover, it is likely that similar long-distance communication mechanisms, as described in our study, are also used to ensure proper assembly and functional regulation of other large ribonucleoprotein complexes.

## Methods

### Yeast strains and plasmids

*Saccharomyces cerevisiae* strains used in this study are listed in Supplementary Table 2 and are, unless otherwise specified, derived from W303. Strains were constructed utilizing established gene disruption, C-terminal tagging, and tetrad dissection methods. For Y2H analyses, the reporter strain PJ69-4A (Supplementary Table 2) was used. Plasmids used in this study are listed in Supplementary Table 3 and were constructed according to standard DNA cloning techniques and verified by sequencing.

### Plasmid shuffle assays

Shuffle strains were constructed by knocking out an essential gene in a diploid yeast strain, transformation with a *URA3* plasmid containing the respective wild-type gene, and sporulation to generate haploids harboring the gene knockout and the complementing *URA3* plasmid. These shuffle strains were transformed with *LEU2* or *TRP1* plasmids carrying different alleles of the gene of interest and analyzed on 5-FOA-containing plates to select for cells that have lost the *URA3* plasmid carrying the wild-type gene, allowing to evaluate the phenotype caused by the allele on the *LEU2* or *TRP1* plasmid. Double shuffle strains contained knockouts of two essential genes complemented by two *URA3* plasmids with wild-type genes, and they were generated by crossing of two different shuffle strains with opposing mating types and subsequent sporulation and identification of haploids containing both knockouts and both *URA3* plasmids with the corresponding wild-type genes. Double shuffle strains were transformed with combinations of *LEU2* and *TRP1* plasmids carrying different alleles of the two genes of interest. They were also analyzed on 5-FOA-containing plates to select for cells that have lost both *URA3* plasmids.

### Y2H interaction analyses

Plasmids expressing the bait proteins, fused to the *GAL4* DNA-binding domain, and the prey proteins, fused to the *GAL4* activation domain, were co-transformed into the reporter strain PJ69-4A. Y2H interactions were documented by spotting representative transformants in ten-fold serial dilution steps on synthetic dextrose complete (SDC)-Trp-Leu, SDC-Trp-Leu-His (*HIS3* reporter), and SDC-Trp-Leu-Ade (*ADE2* reporter) plates. Growth on SDC-Trp-Leu-His plates is indicative of a weak interaction, whereas only relatively strong interactions permit growth on SDC-Trp-Leu-Ade plates.

### Affinity purification of pre-40S particles

Pre-40S particles were purified using the AF Tsr1 as bait protein. Cells expressing C-terminally TAP-tagged Tsr1 (Tsr1-TAP) were grown in 2 L YPD medium at 30 °C and harvested at an OD_600_ of ~1.8. *TSR1*-TAP strains expressing mutant variants of Ltv1 from *LEU2* plasmids were grown in 4 L SDC medium lacking leucine and harvested at an OD_600_ of ~0.9. Cell pellets were resuspended in lysis buffer containing 50 mM TRIS pH 7.5, 100 mM NaCl, 1.5 mM MgCl_2_, 0.075% NP-40, 1 mM DTT, and 1× FY protease inhibitor cocktail (Serva). Cells were broken in the presence of glass beads by shaking in a bead mill (B. Braun homogenizer 853022) cooled with CO_2_. Lysates were cleared by centrifugation at 4 °C for 10 and 30 min at 5000 and 14,000 rpm, respectively. Lysates were incubated with IgG-beads (GE Healthcare) on a rotating wheel at 4 °C for 75 min. Beads were washed with 5 mL lysis buffer w/o protease inhibitors, subsequently transferred into Mobicol columns (Mobitec) and washed with 10 mL lysis buffer. After resuspension in lysis buffer, pre-40S particles were eluted by incubation with TEV protease at room temperature (RT) on a rotating wheel for 90 min. Eluates were analyzed by sodium dodecyl sulfate-polyacrylamide gel electrophoresis (SDS-PAGE) on 4–12% polyacrylamide gels (Invitrogen) and Coomassie staining or western blotting with the indicated antibodies.

### In vitro phosphorylation assay with pre-40S particles

After IgG incubation and subsequent washing steps, bound Tsr1-TAP particles derived from different mutant strains were divided into two Mobicol columns and incubated on a rotating wheel, in the presence or absence of 1 mM ATP, for 30 min at 4 °C with buffer containing 50 mM TRIS pH 7.5, 100 mM NaCl, 5 mM MgCl_2_, 0.075% NP-40, and 1 mM DTT. After washing with 2 mL buffer to remove ATP, TEV-cleavage was performed as described above and eluates were analyzed by SDS-PAGE with 4–12% polyacrylamide gels (Invitrogen) and western blotting.

### ATPase activity measurements on purified pre-40S particles

The corresponding yeast cells (1 L) were grown in YPD supplemented with 20 mg/L adenine at 30 °C to an OD_600_ of 1.5 and harvested by centrifugation. Cell pellets were resuspended in a buffer containing 50 mM Tris-HCl, pH 7.5, 100 mM NaCl, 10 mM MgCl_2_, and 5% glycerol and lysed using a Precellys cell disruptor (Bertin instruments) in 15-ml vials containing 5 mL zirconia beads (*Ø* 100 µm—Roth). Lysis settings were: 6 × 30 s at 6000 rpm with 30 s pause in between; cell disruptor pre-cooled down with liquid nitrogen. Cell lysates were clarified by centrifugation (5 min at 4000 rpm, 4 °C). IgG-beads (GE Healthcare) were added to the resulting clarified supernatants and incubated for 90 min at 4 °C under mild agitation. The immobilized pre-40S particles were first extensively washed in batch (40 beads volumes) and then on a gravity-flow chromatography column (60 beads volumes). TEV protease cleavage was performed overnight (O/N) at 4 °C in the presence of ribonuclease inhibitor. The resulting TEV eluates were stored at 4 °C until used for ATPase assays. The levels of co-purified Rio2 present in the eluates was estimated by western blot analyses and accordingly adjusted in order to use similar amounts of Rio2 in the single turnover assay. For single turnover analysis, 5% of the Rio2-adjusted eluates were first incubated 10 min at 30 °C and then mixed with an equal volume of a pre-warmed γ^32^P-labeled ATP (Hartmann Analytic 6000 Ci/mmol)/ATP mix (750 nCi of γ32P-labeled ATP; ATP end concentration 200 nM). Aliquots (5%) were collected and stopped at the indicated time points by addition of 18 volumes of perchloric acid (1 M) followed by addition of 6 volumes of potassium acetate (8 M) and stored in liquid nitrogen. Reactions were centrifuged at 14,000 rpm for 10 min at RT. Aliquots (1.6%) of each time point were loaded on Polygram Cel 300 PEI (Macherey-Nagel) thin layer chromatography (TLC) plates and developed with 350 mM KH_2_PO_4_ buffer for 45–60 min and dried^14,30^. TLC plates were exposed to a phosphorimager screen and signals were acquired on a Typhoon FLA-9500 (GE Healthcare). Single-turnover experiment quantifications (Image J) and standard deviations were derived from at least two biological replicates (two different purifications) and two technical replicates (two single turnover experiments per purification). Values are expressed relative to the amounts of P_i_ liberated in one wild-type condition after 60 min (set to 100%).

### Western blotting

Western blot analysis was performed using the following antibodies: anti-Rps3 antibody (1:30,000; provided by Matthias Seedorf), anti-Nob1 antibody (1:5000; provided by David Tollervey), anti-Ltv1 antibody (1:8000; provided by Katrin Karbstein), anti-Enp1 antibody (1:4000; provided by Katrin Karbstein), anti-Tsr1 antibody (1:4000; provided by Katrin Karbstein), anti-Dim1 antibody (1:4000; provided by Katrin Karbstein), anti-Pno1 antibody (1:2000; provided by Katrin Karbstein), anti-Rio2-antibody (1:5000; provided by Katrin Karbstein), anti-Hrr25 antibody (1:5000; provided by Wolfgang Zachariae), anti-CBP antibody (1:5000; Merck-Millipore, Cat.Nr. 07-482), secondary anti-rabbit horseradish peroxidase-conjugated antibody (1:15000; Sigma-Aldrich, Cat.Nr. A7058), horseradish-peroxidase-conjugated anti-HA antibody (1:5000; Roche, Cat.Nr. 12013819001). Uncropped scans of the most important blots are provided as a Source Data File.

### Polysome profile analyses

Cells expressing wild-type *RPS20* or the indicated *rps20* mutants were grown at 25 °C in 80 mL of YPD medium to logarithmic growth phase (OD_600_ of ~0.6). In all, 100 μg/mL cycloheximide was added to the cultures, and after incubation for 5 min on ice, cells were pelleted and washed with lysis buffer (10 mM TRIS, pH 7.5, 100 mM NaCl, 30 mM MgCl_2_, 100 μg/mL cycloheximide). After resuspension in lysis buffer and cell lysis with glass beads, 6 *A*_260_ units of the cell extracts were loaded onto 7–45% sucrose gradients (sucrose dissolved in 50 mM TRIS pH 7.5, 50 mM NaCl, 10 mM MgCl_2_) and centrifuged at 180,000 × *g* for 2 h 45 min at 4 °C. Gradients were analyzed using a UA-6 system (Teledyne ISCO) by continuously monitoring at *A*_254_.

### Northern blot analyses

Cells expressing wild-type *RPS20* or the indicated *rps20* mutants were grown in YPD medium at 25 °C to logarithmic growth phase. Cells were resuspended in lysis buffer containing 10 mM TRIS pH 7.5, 10 mM EDTA, and 0.5% SDS and lysed by vortexing in the presence of glass beads. RNA was extracted in two subsequent steps with phenol:chloroform:isoamyl alcohol (25:24:1) and once with chloroform:isoamyl alcohol (24:1). RNA was precipitated by addition of 1/10 volume of 3 M sodium acetate (pH 5.2) and 2.5 volumes of 100% ethanol and, after drying, dissolved in nuclease-free water. Three µg of the isolated RNA was denatured at 65 °C, separated on 1.5% agarose gels (containing 20 mM 3-(*N*-morpholino)propanesulfonic acid (MOPS), 5 mM sodium acetate, 1 mM EDTA, 0.75% formaldehyde, pH 7.0) and transferred O/N onto Hybond N^+^ nylon membranes (GE Healthcare) by capillary transfer. Hybridization with ^32^P 5′-radiolabeled oligonucleotides (probe D/A_2_ (20S): 5′-GACTCTCCATCTCTTGTCTTCTTG-3′, probe 18S: 5′-GCATGGCTTAATCTTTGAGAC-3′, probe 25S: 5′-CTCCGCTTATTGATATGC-3′) was performed O/N at 42 °C in buffer containing 0.5 M Na_2_HPO_4_, pH 7.2, 7% SDS, and 1 mM EDTA. After three subsequent washing steps with buffer containing 40 mM Na_2_HPO_4_, pH 7.2, 1% SDS, signals were detected by exposing X-ray films.

### Fluorescence in situ hybridization, fluorescence microscopy

For fluorescence in situ hybridization^44^, yeast cells were grown in 50 mL YPD at 25 °C to logarithmic growth phase. Cells were fixed by addition of formaldehyde (4% final concentration) for 15 min at RT. Cells were collected by centrifugation, resuspended in 5 ml of 0.1 M KPO_4_ (pH 6.4)/4% formaldehyde, and incubated for 60 min. Subsequently, cells were washed twice with 0.1 M KPO_4_ (pH 6.4), and once with wash buffer (0.1 M KPO_4_ (pH 6.4), 1.2 M sorbitol). The pellet was resuspended in 1 mL wash buffer containing 500 µg/mL zymolyase 100 T (amsbio) for ~30 min at 30 °C, followed by one wash with wash buffer. The spheroblasts were resuspended in ~2-fold pellet volume, applied to adhesive 10-well microscopic slides (Thermo Scientific), and incubated for 10 min. Nonadhered cells were removed by aspiration and washed with 2× SSC (0.3 M NaCl, 0.03 M Na-citrate). Approximately 0.4 pmoles/µL of a Cy3-labeled ITS1-specific probe (5′-Cy3-ATGCTCTTGCCAAAACAAAAAAATCCATTTTCAAAATTATTAAATTTCTT-3′) in 12 µL of hybridization buffer (50% formamide, 10% dextran sulfate, 4× SSC, 0.02% polyvinyl pyrrolidone, 0.02% bovine serum albumin, 0.02% Ficoll-400, 125 µg/mL tRNA, 500 µg/mL of denatured salmon sperm DNA) were applied to each well. The hybridizations were incubated O/N at 37 °C in a humid chamber and subsequently washed at RT for 10 min in 2× SSC, 10 min in 1× SSC, and 10 min in 0.5× SSC. Slides were incubated with 0.5× SSC containing 25 µg/mL DAPI for 5 min and washed two times 10 min in 0.5× SSC. Slides were mounted with Mowiol (Sigma).

Cells were examined by fluorescence microscopy on a Zeiss Axioskop microscope. Live yeast cells expressing GFP fusion proteins were imaged by fluorescence microscopy using a Zeiss Axioskop microscope.

### Pre-40S particle purification for cryo-EM

Mutant *rps20*Δloop cells were grown in 10 L YPD medium at 30 °C to an OD_600_ of ~1.8. Pre-40S particles were purified via Tsr1-TAP as described above (see section: “Affinity purification of pre-40S particles”) but using a lysis buffer containing 5 mM MgCl_2_. After affinity purification, eluted Rps20Δloop pre-40S particles were deposited on a 10–30% sucrose gradient in lysis buffer. Eleven-mL-containing gradient tubes were subjected to ultracentrifugation on a Beckman Coulter Optima L-100 XP Ultracentrifuge, using a SW41 rotor (3 h 20 min, 38,000 rpm, 4 °C). Gradient fractions were subsequently scanned at *A*_254_ and collected using a Fraction Collector system (Foxy R1, Teledyne Isco). Fractions of interest were pooled and sucrose was removed by five successive series of concentrating/washing with lysis buffer on Vivacon 2 centrifugal devices (Sartorius).

### Grid preparation and cryo-EM image acquisition

Cryo-EM grids were prepared and systematically checked at METI, the Toulouse cryo-EM facility. In all, 3.5 µL of purified pre-40S particles (with RNA concentrations ranging from 35 to 45 ng/µL as estimated by NanoDrop measurements) were deposited onto freshly glow-discharged holey carbon grids (QUANTIFOIL^®^ R2/1, 300 mesh with a 2-nm continuous layer of carbon on top). Grids were plunge-frozen using a Leica EM-GP automat; temperature and humidity level of the loading chamber were maintained at 20 °C and 95%, respectively. Excess of solution was blotted with a Whatman filter paper no.1 for 1.7–1.9 s and grids were immediately plunged into liquid ethane (−183 °C).

Cryo-EM images of Tsr1-TAP Rps20Δloop pre-40S particles were recorded on the CM01 beamline at ESRF, Grenoble. The CM01 Titan Krios electron microscope (FEI, ThermoFisher Scientific) was operating at 300 kV and was equipped with a Gatan K2 summit direct electron detector (counting mode). Automatic image acquisition was performed with EPU, at a magnification corresponding to a calibrated pixel size of 1.07 Å and a total electron dose of 32.4 *e*^*−*^/Å^2^ over 25 frames. Defoci values ranged from −0.8 to −3 µm.

### Single particle analysis

A total number of 6480 stacks of frames was collected at ESRF. Frame stacks were aligned to correct for beam-induced motion using MOTIONCOR2^45^. Contrast Transfer Function (CTF) and defocus estimation was performed on the realigned stacks using CTFFIND4^46^. After selection upon CTF estimation quality, maximum resolution on their power spectra, and visual checking, 6041 micrographs were retained for further analysis. In all, 645,109 particles were automatically picked, then extracted in boxes of 384 × 384 pixels, using the RELION 2.1 autopick option. All subsequent image analysis was performed using RELION 2.1^47^ (Supplementary Fig. 7). A first 2D classification was performed (on particle images binned by a factor of 8) to sort out ill-picked particles. The 344,959 remaining particles were binned by a factor of 4 and subjected to a 3D classification in 4 classes, using the 40S subunit extracted from the crystal structure of the *S. cerevisiae* ribosome (PDB 4V88)^29^, low-pass filtered to 60 Å, as initial reference. This resulted in two classes with full 40S morphology and good level of details. Particles from these classes were grouped and re-extracted without imposing any binning factor, and a consensus 3D structure was obtained using 3D auto-refinement in RELION, with an overall resolution of 3.1 Å for FSC = 0.143 according to gold-standard FSC procedure. As the head of this consensus structure harbored highly blurred features, the 171,807 particles grouped in this reconstruction were further submitted to focused 3D classifications around the head region, with signal subtraction to remove information coming from the body of the pre-40S particles, according to ref. ^37^. A group of 54,130 particles (C1-Head only) was obtained from the focused classification around the head region and subsequently auto-refined (using the solvent-flattened FSC option) and post-processed/sharpened to a resolution of 3.75 Å according to RELION’s Gold Standard FSC criterion. Detector MTF correction and global map sharpening was performed using RELION’s post-processing option, with an automatically estimated *B*-factor of −80 Å^2^. Alternatively, local *B*-factor estimation and subsequent local sharpening of the EM map was performed using LocScale^48^ implemented in the CCP-EM software suite^49^. Similarly, 42,901 particles from two 3D classes harboring very similar features were grouped into a so-called C2-Head only class and auto-refined and post-processed together. This yielded a 3D reconstruction solved to 3.75 Å resolution and a *B*-factor of −81 Å^2^.

After this focused classification, particles corresponding to the C1- and C2-Head only classes were retrieved from the dataset without signal subtraction and submitted to auto-refinement and post-processing, yielding maps of full C1-S20Δloop and C2-S20Δloop particles solved to 3.47 and 3.79 Å, respectively (and automatically estimated *B*-factors of −66 and −83 Å^2^, respectively). Local *B*-factor estimation was performed using LocScale^48,49^.

Similarly, the consensus structure was subjected to focused classification with signal subtraction followed by auto-refinement and post-processing around the lower part of the platform, yielding a 3D reconstruction called Dim1 solved to 3.15 Å resolution and with a *B*-factor of −40 Å^2^. For all the cryo-EM maps described above, estimation of local resolution was performed with the ResMap software^50^.

### Cryo-EM Map interpretation

Atomic models of pre-40S particles (PDB 6EML, 6FAI)^16,20^ or the mature 40S subunit (PDB 4V88)^29^ were first fitted into the 3D maps of interest as rigid body using the fit command in UCSF Chimera^51^. Heads were modeled separately in the C1- and C2-Head only maps and then adapted to the corresponding full maps to generate whole atomic models using UCSF Chimera and Coot^52^. For the C1-S20Δloop structure, map segmentation was realized with the Segger option in Chimera, and the X-ray structure of the Yar1-bound form of Rps3 (PDB 4BSZ)^39^ was rigid-body fitted into the segmented region of interest. A similar approach was used to fit the X-ray structure of Hrr25 into the density attributed to factor X in the C2-Head only map, as well as the 3D structure of Dim1 into the Dim1 cryo-EM map. The yeast 3D structure of Dim1 was first modeled from the X-ray structure of its human counterpart (PDB 1ZQ9) using I-Tasser^53^. Manual refinements and adjustments, as well as flexible and jiggle fittings were then realized on various chains of the whole models in Coot^54^. The two-nucleotide extension of the rRNA ITS1 was built in its density in the C2-S20Δloop full map with Coot.

Final atomic models were refined using REFMAC5^55^ and Phenix_RealSpace_Refine^56^, with secondary structure restraints for proteins and RNA generated by ProSMART^57^ and LIBG^54^. Final model evaluation was done with MolProbity^58^. Overfitting statistics were calculated by a random displacement of atoms in the model, followed by a refinement against one of the half-maps in REFMAC5, and Fourier shell correlation curves were calculated between the volume from the atomic model and each of the half-maps in REFMAC5 (Supplementary Table 1).

Map and model visualization was done with Coot and UCSF Chimera; figures were created using UCSF Chimera.

### Reporting summary

Further information on research design is available in the Nature Research Reporting Summary linked to this article.

## Supplementary information

Supplementary Information

Peer Review File

Reporting Summary

Source Data

## Data Availability

Cryo-EM maps have been deposited in the Electron Microscopy Data Bank (EMDB), under the accession codes: EMD-4792 (C1-S20Δloop), EMD-4793 (C2-S20Δloop), EMD-4794 (C1-Head only), EMD-4795 (C2-Head only), EMD-4796 (Dim1). Two atomic coordinate models have been deposited in the PDB with accession codes 6RBD and 6RBE, corresponding to the C1-S20Δloop and C2-S20Δloop maps, respectively. The source data underlying Fig. 4 are provided as a Source Data file.
