# Supplementary material for: Conformational proofreading of distant 40S ribosomal subunit maturation events by a long-range communication mechanism
Source: Nat Commun. 2019 Jun 21;10:2754. doi: 10.1038/s41467-019-10678-z (PMC6588571; doi:10.1038/s41467-019-10678-z)
Supplement: Supplementary file 1 — Supplementary Information [file 41467_2019_10678_MOESM1_ESM.pdf]

Supplementary Information

Conformational proofreading of distant 40S ribosomal subunit maturation events by a long-range communication mechanism

Mitterer et al., 2019

Supplementary Figure 1

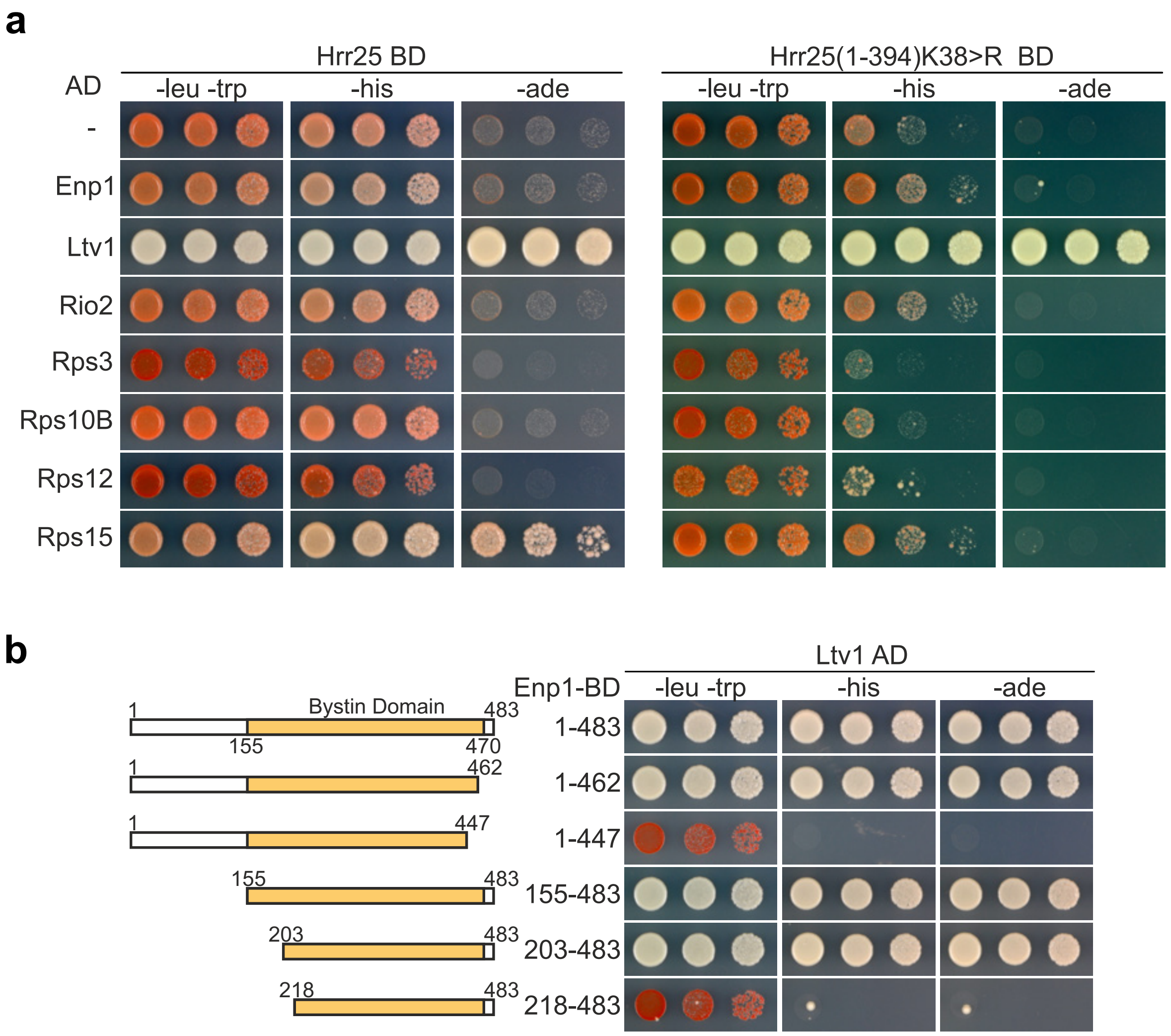

Supplementary Figure 1. Interaction of Ltv1 with Hrr25 and Enp1. **a** Ltv1 is the main interaction partner of Hrr25 on the pre-40S particle. Full-length Hrr25, fused to the Gal4 DNA-binding domain (Hrr25-BD), was tested for Y2H interaction with the depicted AFs and r-proteins located at the head of the 40S subunit, fused to the Gal4 activation domain (AD). Cells were spotted in 10-fold serial dilutions on SDC-Leu-Trp, SDC-His-Leu-Trp (-his; growth on this medium indicates a weak interaction), and SDC-Ade-Leu-Trp (-ade; growth on this medium indicates a strong interaction) plates. Note that full-length Hrr25 shows some self-activation of the *HIS3* reporter gene (left panel). The interactions were also tested using a C-terminally truncated Hrr25 variant (1-394) carrying additionally an exchange of a catalytically important residue (K38R), which showed almost no self-activation (right panel). Note that Ltv1 was the only protein showing a strong interaction with Hrr25 in both conditions. A weaker interaction was observed between Hrr25 and Rps15, which was however lost with the truncated Hrr25 variant. **b** The bystin domain of Enp1 mediates the interaction with Ltv1. The indicated Enp1 Gal4 DNA-binding domain fusions (Enp1-BD) were tested for Y2H interaction with an Ltv1 Gal4 activation domain fusion (Ltv1-AD). See **a** for a detailed description.

Supplementary Figure 2

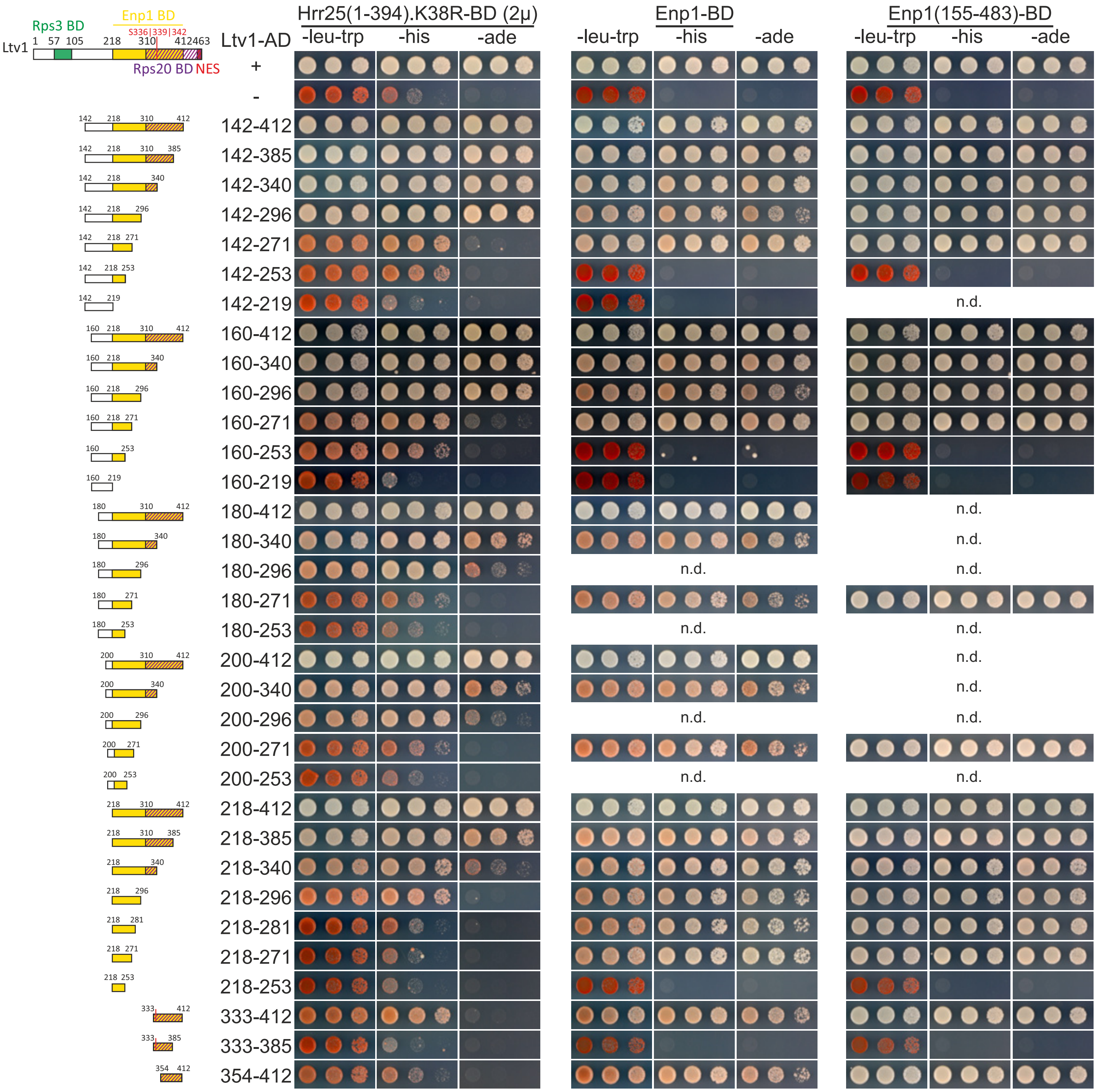

Supplementary Figure 2. Ltv1 has two minimal, largely overlapping Hrr25 and Enp1 binding sites. The indicated Ltv1 truncation variants, fused to the Gal4 activation domain (Ltv1-AD), were tested for interaction with Hrr25(1-394).K38R, full-length Enp1, and Enp1.155C (amino acids 155-483), fused to the Gal4 DNA-binding domain (BD). See legend of Supplementary Fig. 1 for a detailed description.

Supplementary Figure 3

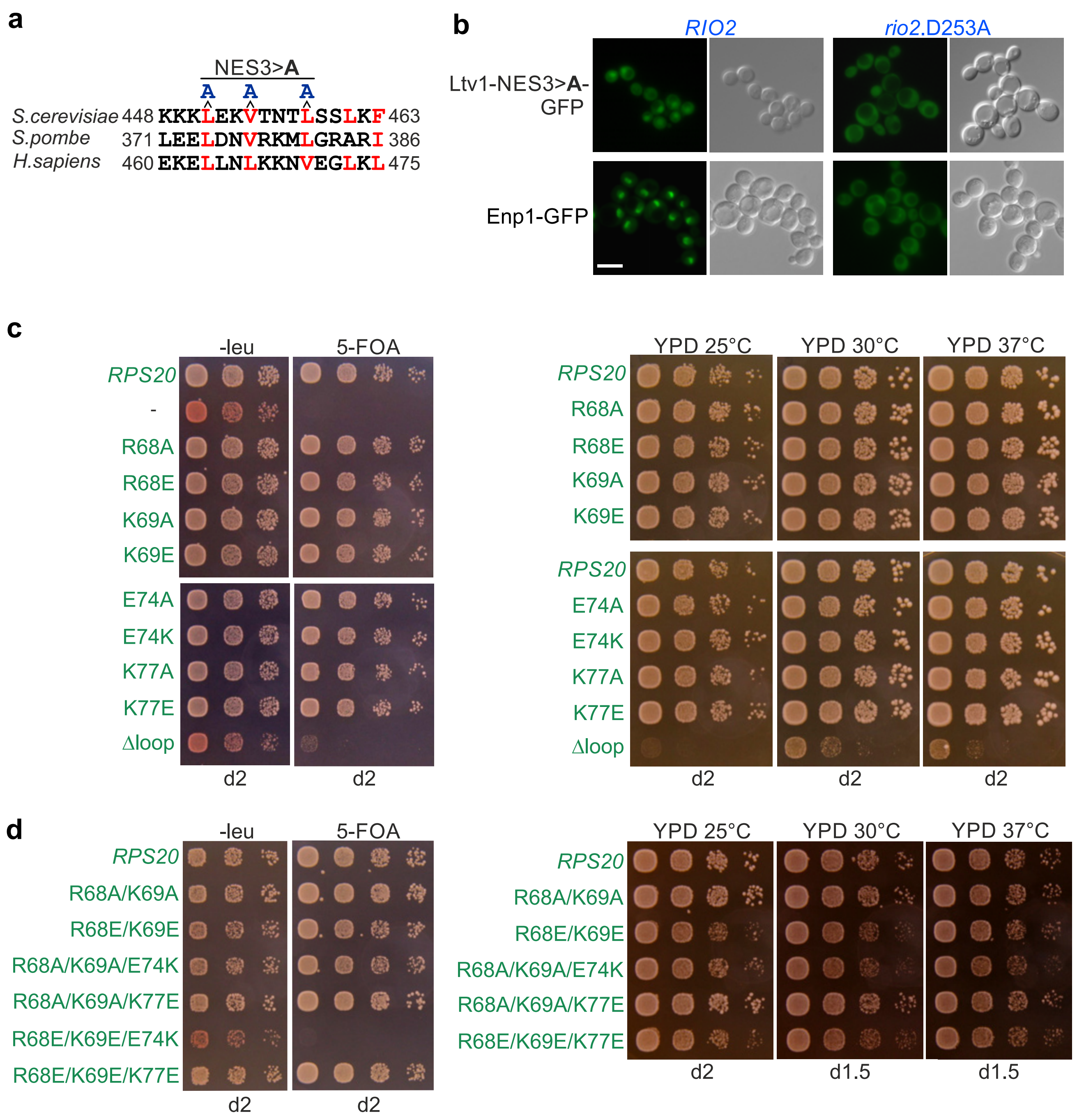

Supplementary Figure 3. Localization of Ltv1 and Enp1 in *rio2*.D253A mutants and growth phenotype of *rps20* loop mutants. **a** Alignment of the C-terminal sequence of Ltv1. The amino acids comprising its nuclear export sequence (NES) are marked in red and the three exchanges in the Ltv1-NES3>**A** reporter construct are indicated. **b** The subcellular localization of Enp1-GFP and the Ltv1-NES3>**A**-GFP reporter construct (note that the point mutations in the C-terminal NES of Ltv1 lead to a predominantly nuclear steady-state localization of the protein), was assessed by fluorescence microscopy in cells expressing wild-type *RIO2* or the catalytically inactive *rio2*.D253A allele, revealing that recycling of these AFs back to the nucleus relies on the ATPase activity of Rio2. Scale bar is 5 µm. **c, d** An *RPS20* (*rps20*Δ) shuffle strain was transformed with plasmid-based wild-type *RPS20* or the indicated *rps20* mutant alleles. Representative transformants were spotted in 10-fold serial dilutions on SDC-Leu and SDC+5-FOA plates and incubated at 30°C for 2 days (left panels). After plasmid shuffling on 5-FOA, strains were spotted in 10-fold serial dilutions on YPD plates and incubated at the indicated temperatures for 1.5 or 2 days (right panels).

Supplementary Figure 4

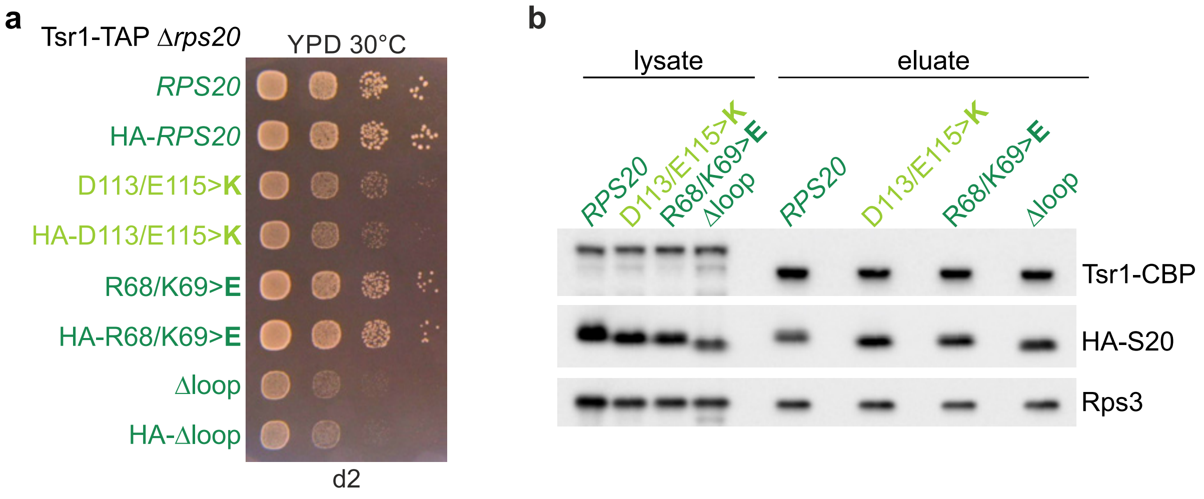

Supplementary Figure 4. Rps20 variants are incorporated into pre-40S particles. **a** An *RPS20* (*rps20*Δ) shuffle *TSR1*-TAP strain was transformed with plasmid-based wild-type *RPS20* or the indicated *rps20* mutant alleles, either untagged or fused to an N-terminal HA-tag. After plasmid shuffling on 5-FOA-containing plates, cells were spotted on YPD plates and incubated at 30°C for 2 days. Note that N-terminal HA-tag fusion had no effect on growth, indicating the tag does not disturb the function of Rps20. **b** Tsr1-TAP particles were isolated from cells expressing the indicated Rps20 variants, fused to an N-terminal HA-tag, and analyzed by SDS-PAGE and Western blotting (lysates and eluates). Note that all HA-Rps20 variants were incorporated into pre-40S particles.

Supplementary Figure 5

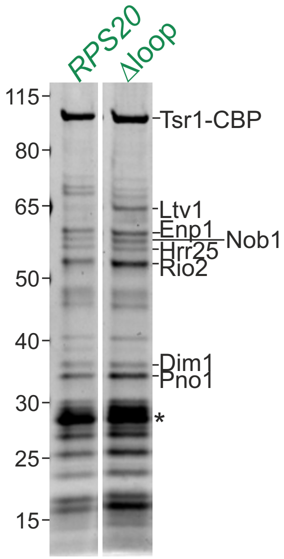

Supplementary Figure 5. Accumulation of AFs in S20Δloop pre-40S particles. Tsr1-TAP particles were isolated from cells expressing either wild-type *RPS20* (left lane) or the *rps20*Δloop mutant allele (right lane) and analyzed by SDS-PAGE and Coomassie staining. The asterisk indicates the band of the TEV protease, which was used to elute pre-40S particles from the IgG beads.

Supplementary Figure 6

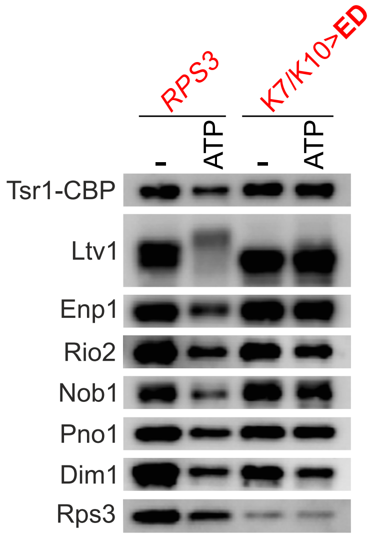

Supplementary Figure 6. The rps3.K7/K10>**ED** N-domain-assembly mutant prevents Ltv1 phosphorylation and release. Tsr1-TAP particles were isolated from cells expressing wild-type *RPS3* or the *rps3*(K7/K10>**ED**) mutant allele and the *in vitro* phosphorylation assay was performed as described in Fig. 4. Eluates were analyzed by Western blotting using the indicated antibodies. Note that, because the used Rps3 antibody was raised against a short N-terminal epitope of Rps3 including lysines K7 and K10, Rps3 detection is impaired in samples derived from the *rps3*.K7/K10>**ED** mutant.

Supplementary Figure 7

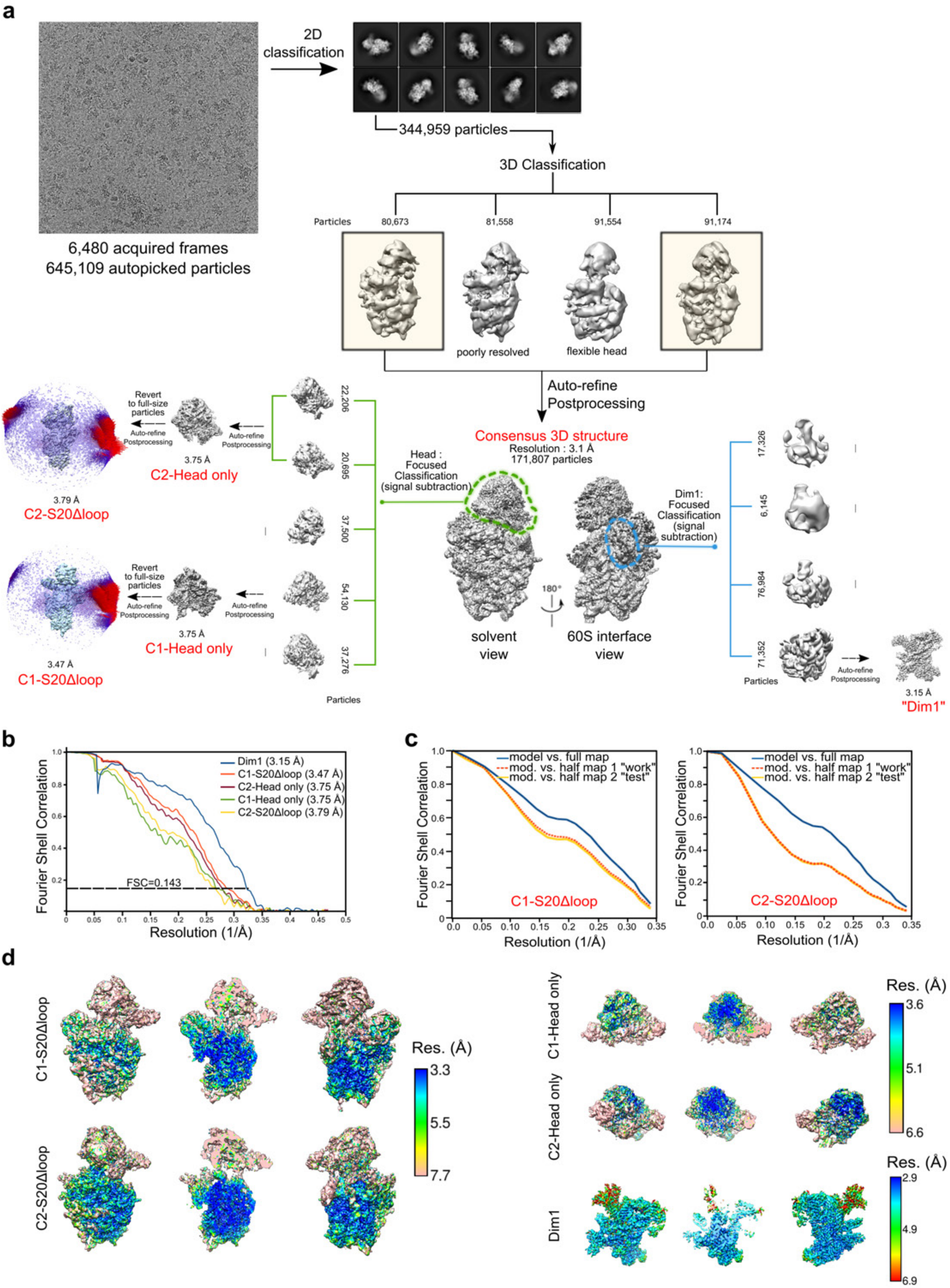

Supplementary Figure 7: Cryo-EM image processing scheme, and cryo-EM maps and model validations. **a** Single particle analysis strategy applied for obtaining the C1- and C2-S20Δloop structures. Final full C1- and C2-S20Δloop maps are presented as viewed from the beak, with angular coverage for both map reconstructions. Spike heights are proportional to orientation occurrences. **b** Gold standard FSC curves for the various cryo-EM maps obtained. **c** Validation of the atomic models derived from the cryo-EM maps of C1-S20Δloop (left panel) and C2-S20Δloop (right panel) pre-40S particles, as calculated by REFMAC5 (see Supplementary Table 1 for model refinement details). **d** Local resolutions of the different cryo-EM maps shown in **a**, as estimated by ResMap[1]. For all C1- and C2-S20Δloop maps, left panels represent surface views as seen from the solvent side, middle panels are cutaway views from the same side, and right panels are surface views seen from the 60S interface. For the "Dim1" map, the left panel is a surface view as seen from the 60S interface, the middle panel is a cutaway view with the same orientation, and the right panel is a surface view seen from the solvent side of the particle.

Supplementary Figure 8

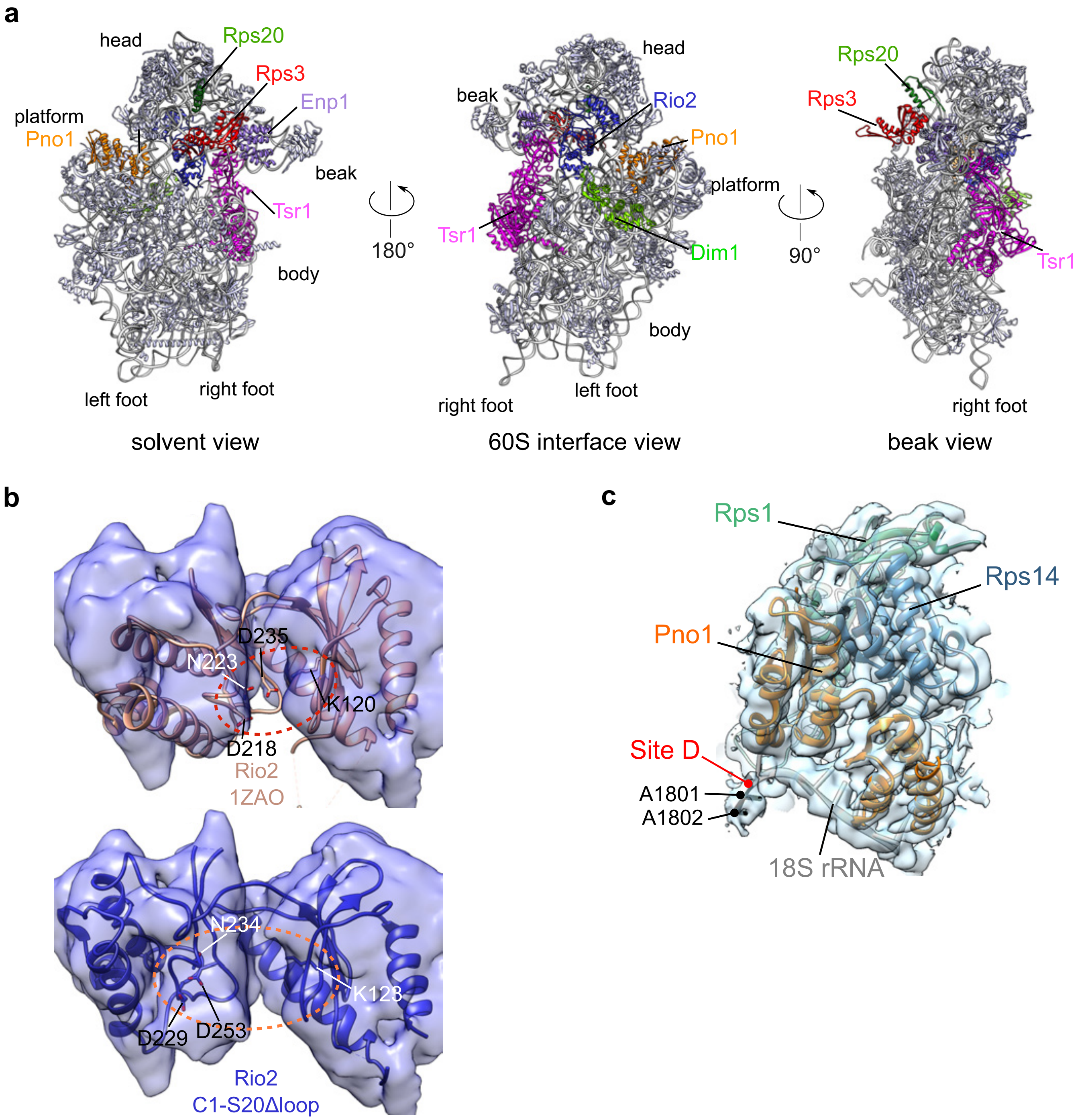

Supplementary Figure 8. Overview and details of the structure of C1-S20Δloop pre-40S particles. **a** Atomic model of C1-S20Δloop pre-40S particles viewed from the solvent side (left panel), 60S interface (middle panel), and the beak (right panel). AFs and r-proteins of interest have been colored as indicated in the model, other r-proteins are displayed in pale blue, rRNA in grey. **b** Density attributed to Rio2 segmented from the C1-S20Δloop cryo-EM map (transparent blue surface), fitted either with the X-ray structure of ATP-bound Rio2 from *A. fulgidus* (PDB 1ZAO)[2] (upper panel) or the C1-S20Δloop modeled Rio2. The catalytic pocket is indicated by a dotted ellipsoid and catalytically important residues are depicted, revealing the opening of the ATP-binding domain of Rio2 in C1-S20Δloop pre-40S particles. **c** Details of the platform region of C1-S20Δloop pre-40S particles. Cryo-EM density surface is shown in pale blue. rRNA is shown in grey and the two nucleotides (A1801, A1802) following cleavage site D, which are distinguished in the EM density, are indicated. Pno1, Rps14, and Rps1 are shown in orange, blue, and green, respectively.

Supplementary Figure 9

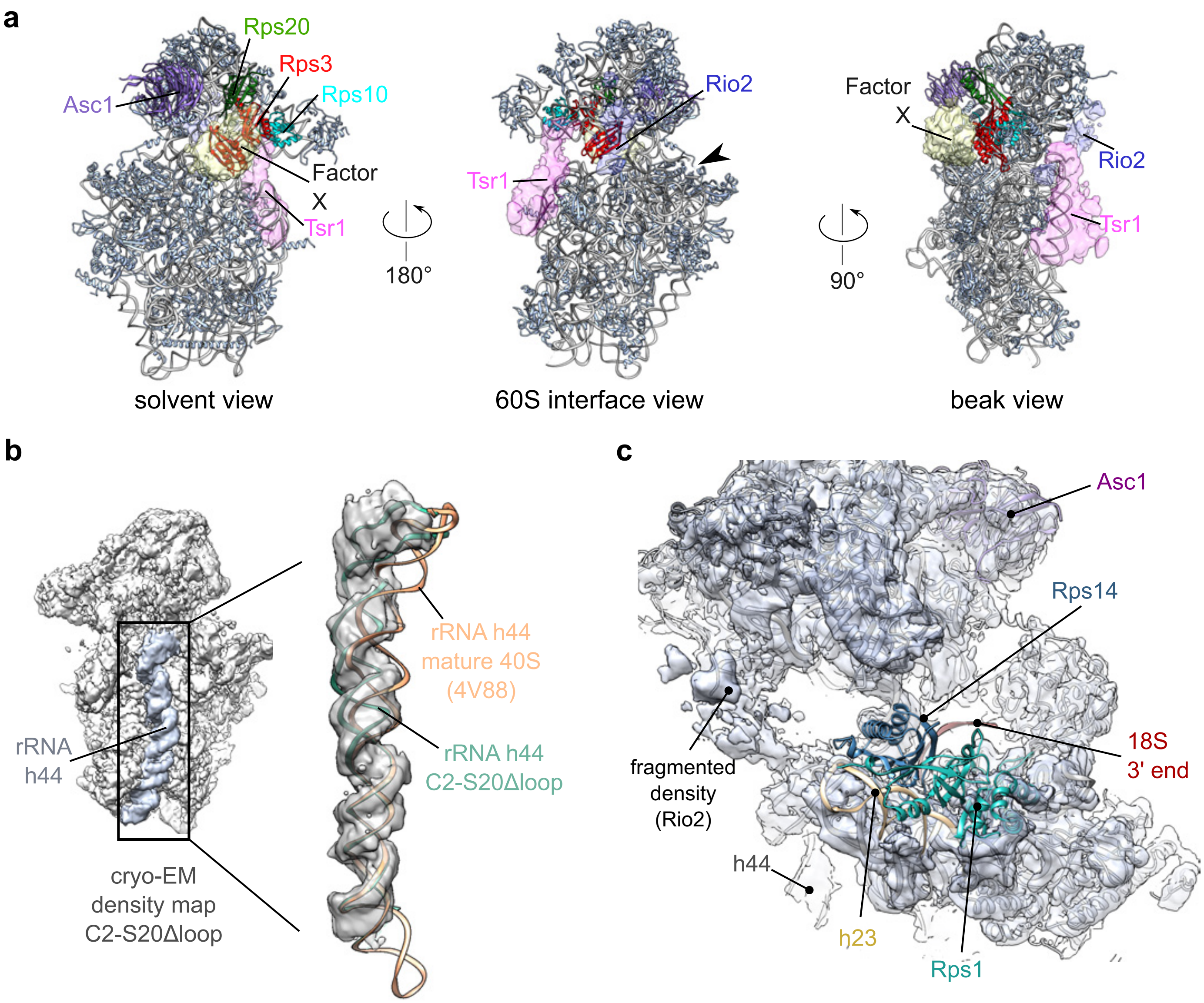

Supplementary Figure 9: Overview and details of the structure of C2-S20Δloop pre-40S particles. **a** Atomic model of C2-S20Δloop pre-40S particles viewed from the solvent side (left panel), 60S interface (middle panel), and the beak (right panel). AFs and r-proteins of interest have been colored as indicated in the model, other r-proteins are displayed in pale blue, rRNA is displayed in grey. Low-resolution densities attributed to Rio2, Tsr1, and unidentified Factor X (resembling factor X described in[3]) have been segmented from the cryo-EM map and are indicated but have not been modeled in the C2-S20Δloop atomic model. **b** rRNA helix h44 of C2-S20Δloop pre-40S particles is in an immature position. The cryo-EM density map corresponding to this helix has been segmented; the atomic model of rRNA h44 in C2-S20Δloop is represented in cyan, and rRNA h44 as found in the mature 40S subunit (PDB 4V88)[4] is in orange. **c** Platform view of C2-S20Δloop pre-40S particles. The cryo-EM density is in transparent grey, revealing that Rps1, Rps14, and rRNA helix h23 and the 18S rRNA 3' end cannot be fitted into it, suggesting high dynamics of the platform region.

Supplementary Figure 10

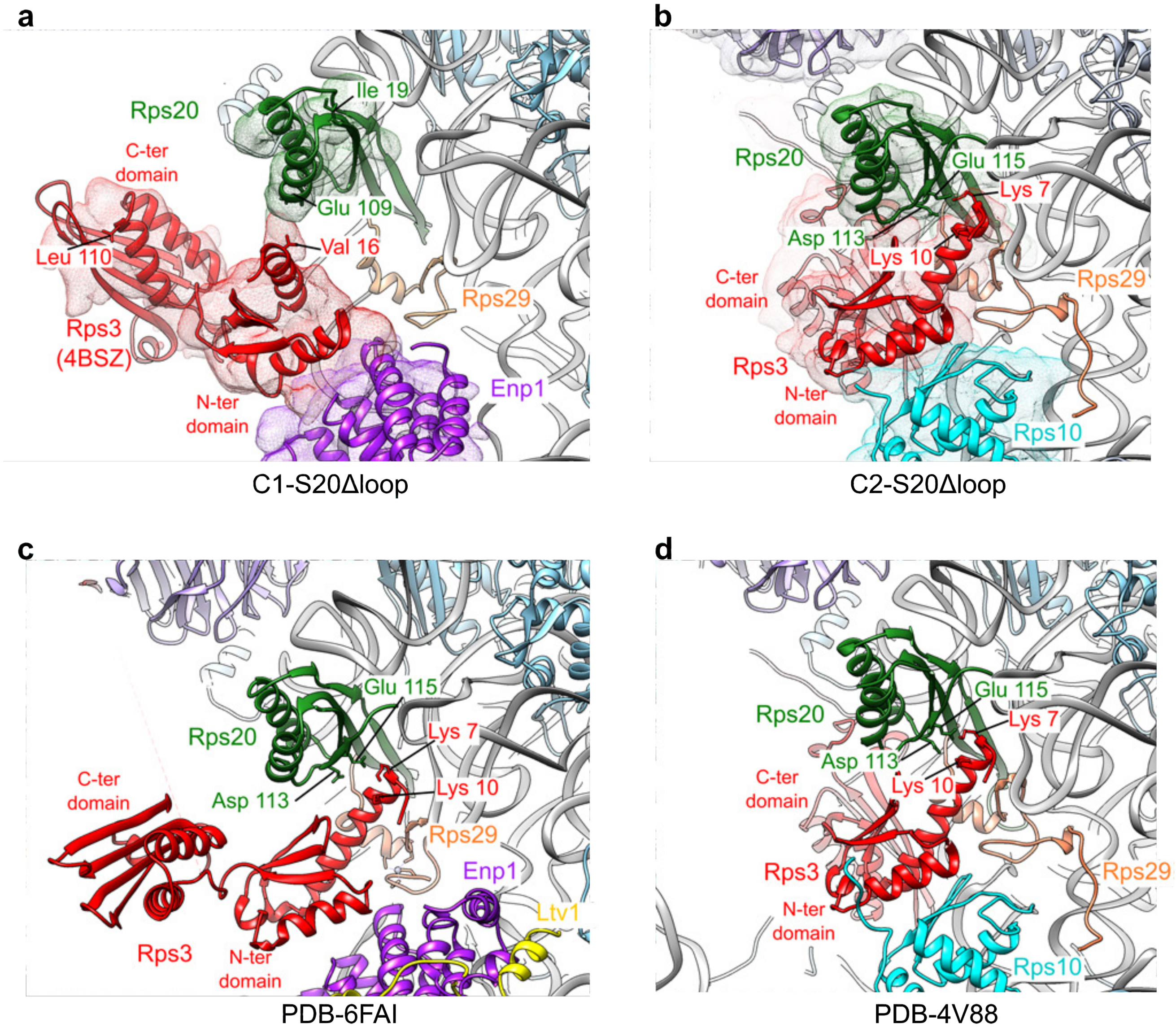

Supplementary Figure 10. Comparison of the positioning of Rps3 and the beak region of (pre)-40S subunits in the atomic models of **a**, C1-S20Δloop pre-40S particles, **b**, C2-S20Δloop pre-40S particles, **c**, pre-40S particles purified with a mutant version of Nob1 as bait (PDB 6FAI)[5], and **d**, mature 40S ribosomal subunit (PDB 4V88)[4]. Segmented cryo-EM densities of the C1-S20Δloop and C2-S20Δloop maps corresponding to Rps20, Rps3, Enp1, and Rps10 are represented in green, red, purple, and turquoise, respectively. Some individual residues within the structures are labeled to allow better orientation.

Supplementary Figure 11

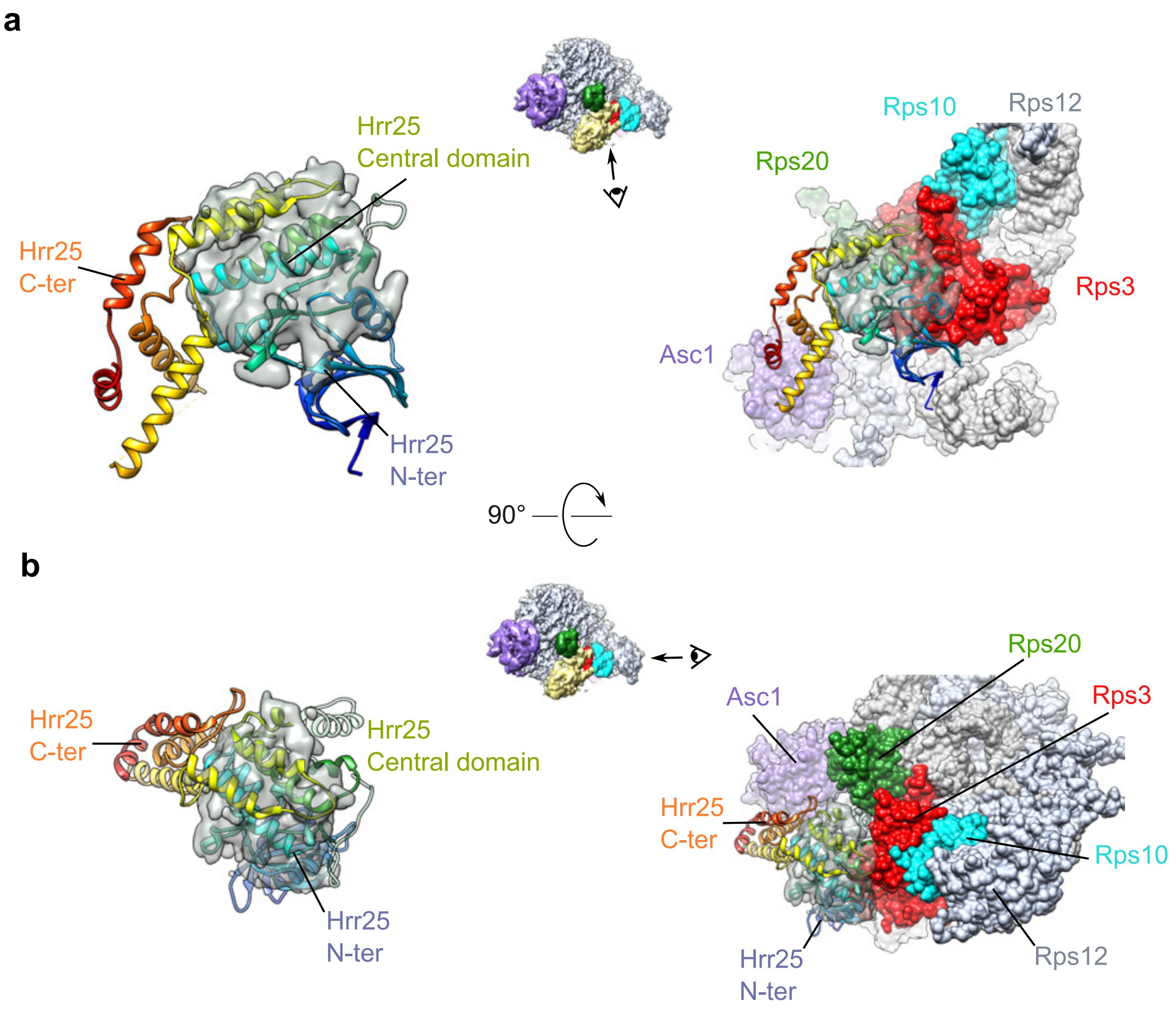

Supplementary Figure 11: Fitting of the factor X cryo-EM density present in the C2-S20Δloop map with the atomic model of Hrr25 (PDB 5CZO)[6]. Left panels represent the cryo-EM density corresponding to factor X, segmented from the C2-Head only map, and fitted with the atomic model of Hrr25 using the "fit to segment" option in Chimera. Right panels represent this fitting in the context of the C2-S20Δloop pre-40S head atomic model, viewed from **a**, under the head and **b**, the beak.

Supplementary Table 1. Cryo-EM data collection, atomic model refinement, and validation statistics.

	C1-S20ΔLoop (EMD-4792) (PDB 6RBD)	C2-S20ΔLoop (EMD-4793) (PDB 6RBE)	C1-Head EMD-4794	C2-Head EMD-4795	Dim1 EMD-4796
**Data collection and processing**					
Magnification	130,000	130,000	130,000	130,000	130,000
Voltage (kV)	300	300	300	300	300
Electron exposure (e–/Å^2)	32.4	32.4	32.4	32.4	32.4
Defocus range (μm)	0.8 – 3.0	0.8 – 3.0	0.8 – 3.0	0.8 – 3.0	0.8 – 3.0
Pixel size (Å)	1.067	1.067	1.067	1.067	1.067
Symmetry imposed	C1	C1	C1	C1	C1
Initial particle images (no.)	344,959	344,959	344,959	344,959	344,959
Final particle images (no.)	54,130	42,901	54,130	42,901	71,352
Map resolution (Å)	3.47	3.79	3.75	3.75	3.15
FSC threshold	0.143	0.143	0.143	0.143	0.143
Map resolution range (Å)	3.2-7.8	3.2-7.8	3.6-6.6	3.6-6.6	2.9-6.9
**Refinement**					
Initial model used (PDB code)	6FAI	4V88			
Model resolution (Å)	3.81	3.92			
FSC threshold	0.5	0.5			
Model resolution range (Å)					
Map sharpening *B* factor (Å^2)	-66	-83			
Model composition					
Non-hydrogen atoms	81048	72721			
Protein residues	5504	4512			
RNA bases	1777	1750			
B factors (Å^2)					
Protein	229	241			
R.m.s. deviations					
Bond lengths (Å)	0.01	0.01			
Bond angles (°)	0.96	1.23			
Validation					
MolProbity score	1.79	2.21			
Clashscore	7.79	9.15			
Poor rotamers (%)	0.28	1.35			
Ramachandran plot					
Favored (%)	94.63	86.84			
Allowed (%)	5.34	11.92			
Disallowed (%)	0.04	1.24			
Validation (RNA)					
correct sugar puckers (%)	99.04	97.2			
Good backbone conformations	65.28	64.29			

Supplementary Table 2. Yeast strains

Name	Genotype	Source
W303	*ade2-1, his3-11, 15, leu2-3,112, trp1-1, ura3-1, can1-100*	[7]
RIO2 Shuffle	W303 *MAT*α *rio2*::*HIS3*MX4 [pRS316-*RIO2*-GFP]	this study
RIO2 Shuffle Δ*ltv1*	W303 *MAT*α *rio2*::*HIS3*MX4 [pRS316-*RIO2*-GFP] *ltv1*::kanMX4	this study
RIO2 Shuffle *ENP1*-GFP	W303 *MAT*α *rio2*::*HIS3*MX4 [pRS316-*RIO2*-GFP] *ENP1*-GFP::natNT2	this study
RIO2 Shuffle *RPS20* Shuffle	W303 *rps20*::natNT2 *ade3*::kanMX [pHT4467Δ-*RPS20*] *rio2*::*HIS3*MX4 [pRS316-*RIO2*-GFP]	this study
RIO2 Shuffle *RPS3* Shuffle	W303 *rps3*::natNT2 *ade3*::kanMX4 [pHT4467Δ-*RPS3*] *rio2*::*HIS3*MX4 [pRS316-*RIO2*-GFP]	this study
RPS20 Shuffle	W303 *MAT***a** *rps20*::natNT2 *ade3*::kanMX4 [pHT4467Δ-*RPS20*]	[8]
RPS20 Shuffle *TSR1*-TAP	W303 *MAT***a** *rps20*::natNT2 *ade3*::kanMX4 [pHT4467Δ-*RPS20*] *TSR1*-TAP::*HIS3*MX4	this study
RPS20 Shuffle Δ*ltv1*	W303 *MAT***a** *rps20*::natNT2 [pHT4467Δ-*RPS20*] *ltv1*::*HIS3*MX4 *ade3*::kanMX4	[8]
RPS20 Shuffle *RPS3* Shuffle	W303 *MAT*α *rps3*::natNT2 [pHT4467Δ-*RPS3*] *rps20*::*HIS3*MX4 [YCplac33- *RPS20*] *ade3*::kanMX4	[8]
RPS3 Shuffle *TSR1*-TAP	W303 *MAT*α *rps3*::natNT2 [pHT4467Δ-*RPS3*] *TSR1*-TAP::*HIS3*MX4	this study
RIO2 Shuffle *TSR1*-TAP	W303 *MAT*α *rio2*::*HIS3*MX4 [pRS316-*RIO2*-GFP] *TSR1*-TAP::*HIS3*MX4	this study
Δ*ltv1 TSR1-TAP*	W303 *MAT***a** *TSR1*-TAP::*HIS3*MX4 *ltv1*::hphNT1	this study
BY4742	*MAT*α, *his3*Δ1, *leu2*Δ0, *lys2*Δ0, *ura3*Δ0	[9]
PJ69-4A	*trp1-901 leu2-3,112 ura3-52 his3-200 gal4Δ gal80Δ LYS2*::*GAL1-HIS3 GAL2-ADE2 met2*::*GAL7*-lacZ	[10]

Supplementary Table 3. Plasmids

Name	relevant information	source
YCplac111	CEN, *LEU2*	[11]
pRS316-*RIO2*-GFP	CEN, *LEU2*, P*RIO2*, T*RIO2*	[12]
pRS315-*RIO2*	CEN, *LEU2*, P*RIO2*, T*RIO2*	[12]
pRS315-*rio2*.D253A	CEN, *LEU2*, P*RIO2*, T*RIO2*	[13]
pRS314-*RIO2*	CEN, *LEU2*, P*RIO2*, T*RIO2*	this study
pRS314-*rio2*.D253A	CEN, *LEU2*, P*RIO2*, T*RIO2*	this study
YCplac111-*LTV1*	CEN, *LEU2*, P*LTV1*, T*ADH1*	[8]
YCplac111-*ltv1*.S336/S339/S342>**A**	CEN, *LEU2*, P*LTV1*, T*ADH1*	[8]
YCplac22-*LTV1*	CEN, *TRP1*, P*LTV1*, T*ADH1*	this study
YCplac22-*ltv1*.S336/S339/S342>**A**	CEN, *TRP1*, P*LTV1*, T*ADH1*	this study
YCplac111-*ltv1*.NES3A-$(GA)_5$-yEGFP	CEN, *LEU2*, P*LTV1*, T*ADH1*	this study

pHT4467Δ-*RPS3*	CEN6 (instable), *URA3*, *ADE3*, P*RPS3*, T*ADH1*	[8]
YCplac111-*RPS3*	CEN, *LEU2*, P*RPS3*, T*ADH1*	[8]
YCplac111-*rps3*.K7/K10>**ED**	CEN, *LEU2*, P*RPS3*, T*ADH1*	[8]
YCplac22-*RPS3*	CEN, *TRP1*, P*RPS3*, T*ADH1*	[8]
YCplac22-*rps3*.K7/K10>**A**	CEN, *TRP1*, P*RPS3*, T*ADH1*	[8]
YCplac22-*rps3*.K8/R9>**A**	CEN, *TRP1*, P*RPS3*, T*ADH1*	[8]
YCplac22-*rps3*.K7/K10>**ED**	CEN, *TRP1*, P*RPS3*, T*ADH1*	[8]
YCplac22-*rps3*.K7/K8/R9/K10>**A**	CEN, *TRP1*, P*RPS3*, T*ADH1*	[8]
pHT4467Δ-*RPS20*	CEN6 (instable), *URA3*, *ADE3*, P*RPS20*, T*ADH1*	[8]
YCplac111-*RPS20*	CEN, *LEU2*, P*RPS20*, T*ADH1*	[8]
YCplac111-*rps20*.D113/E115>**A**	CEN, *LEU2*, P*RPS20*, T*ADH1*	[8]
YCplac111-*rps20*.D113/E115>**K**	CEN, *LEU2*, P*RPS20*, T*ADH1*	[8]
YCplac111-*rps20*Δ68-78-GAGA (*rps20*Δloop)	CEN, *LEU2*, P*RPS20*, T*ADH1,* amino acids 68-78 replaced by GAGA linker	this study
YCplac111-*rps20*.R68/K69>**A**	CEN, *LEU2*, P*RPS20*, T*ADH1*	this study
YCplac111-*rps20*.R68/K69>**E**	CEN, *LEU2*, P*RPS20*, T*ADH1*	this study
YCplac111-*rps20*.E74K	CEN, *LEU2*, P*RPS20*, T*ADH1*	this study
YCplac111-*rps20*.E74A	CEN, *LEU2*, P*RPS20*, T*ADH1*	this study
YCplac111-*rps20*.R68/K69>**A**/E74K	CEN, *LEU2*, P*RPS20*, T*ADH1*	this study
YCplac111-*rps20*.R68/K69>**E**/E74K	CEN, *LEU2*, P*RPS20*, T*ADH1*	this study
YCplac111-*rps20*.R68E	CEN, *LEU2*, P*RPS20*, T*ADH1*	this study
YCplac111-*rps20*.R68A	CEN, *LEU2*, P*RPS20*, T*ADH1*	this study
YCplac111-*rps20*.K69E	CEN, *LEU2*, P*RPS20*, T*ADH1*	this study
YCplac111-*rps20*.K69A	CEN, *LEU2*, P*RPS20*, T*ADH1*	this study
YCplac111-*rps20*.K77E	CEN, *LEU2*, P*RPS20*, T*ADH1*	this study
YCplac111-*rps20*.K77A	CEN, *LEU2*, P*RPS20*, T*ADH1*	this study
YCplac111-*rps20*.R68/K69>**A**/K77E	CEN, *LEU2*, P*RPS20*, T*ADH1*	this study
YCplac111-*rps20*.R68/K69/K77>**E**	CEN, *LEU2*, P*RPS20*, T*ADH1*	this study
YCplac111-*rps20*.R68/K69>**A**/D113/E115>**K**	CEN, *LEU2*, P*RPS20*, T*ADH1*	this study
YCplac111-*rps20*.R68/K69>**E**/D113/E115>**K**	CEN, *LEU2*, P*RPS20*, T*ADH1*	this study
YCplac111-*rps20*.E74K/D113/E115>**K**	CEN, *LEU2*, P*RPS20*, T*ADH1*	this study
YCplac111-*rps20*.R68/K69>**A**/E74K/D113/E115>**K**	CEN, *LEU2*, P*RPS20*, T*ADH1*	this study
YCplac111-*rps20*Δ68-78-GAGA/D113/E115>**K**	CEN, *LEU2*, P*RPS20*, T*ADH1*	this study
YCplac111-	CEN, *LEU2*, P*RPS20*, T*ADH1*	this study

rps20.R68/K69>**E**/D113/E115>**A**		
YCplac111-HA-*RPS20*	CEN, *LEU2*, P*RPS20*, N-terminal 2xHA, T*ADH1*	this study
YCplac111-HA-*rps20*.D113/E115>**K**	CEN, *LEU2*, P*RPS20*, N-terminal 2xHA, T*ADH1*	this study
YCplac111-HA-*rps20*.R68/K69>**E**	CEN, *LEU2*, P*RPS20*, N-terminal 2xHA, T*ADH1*	this study
YCplac111-HA-*rps20*Δ68-78-GAGA (*rps20*Δloop)	CEN, *LEU2*, P*RPS20*, N-terminal 2xHA, T*ADH1*, amino acids 68-78 replaced by GAGA linker	this study
pGAG4BDC112-*HRR25* *	2µ, *TRP1*, P*ADH1*, T*ADH1*, C-terminal $(GA)_5$-G4BD	this study
pGAG4BDC22-*ENP1* *	CEN, *TRP1*, P*ADH1*, T*ADH1*, C-terminal $(GA)_5$-G4BD	this study
pGAG4ADC111-*ENP1*	CEN, *LEU2*, P*ADH1*, T*ADH1*, C-terminal $(GA)_5$-G4AD	this study
pGAG4ADC111-*LTV1* *	CEN, *LEU2*, P*ADH1*, T*ADH1*, C-terminal $(GA)_5$-G4AD	[8]
pGAG4ADC111-*RIO2*	CEN, *LEU2*, P*ADH1*, T*ADH1*, C-terminal $(GA)_5$-G4AD	this study
pGAG4ADC111-*RPS3*	CEN, *LEU2*, P*ADH1*, T*ADH1*, C-terminal $(GA)_5$-G4AD	this study
pGAG4ADC111-*RPS10B*	CEN, *LEU2*, P*ADH1*, T*ADH1*, C-terminal $(GA)_5$-G4AD	this study
pGAG4ADC111-*RPS12*	CEN, *LEU2*, P*ADH1*, T*ADH1*, C-terminal $(GA)_5$-G4AD	this study
pGAG4ADC111-*RPS15*	CEN, *LEU2*, P*ADH1*, T*ADH1*, C-terminal $(GA)_5$-G4AD	this study
pADH195-*ENP1*	2µ, *URA3*, P*ADH1*, T*ADH1*	this study

P denotes the promotor, T the terminator; * for simplicity, only the yeast two-hybrid (Y2H) plasmids containing the respective wild-type genes are listed. The mutant and deletion variants thereof used in the Y2H were cloned into the listed plasmids.

SUPPLEMENTARY REFERENCES

1. Kucukelbir, A., Sigworth, F. J. & Tagare, H. D. Quantifying the local resolution of cryo-EM density maps. *Nat. Methods* **11**, 63–65 (2014).
2. LaRonde-LeBlanc, N., Guszczynski, T., Copeland, T. & Wlodawer, A. Autophosphorylation of Archaeoglobus fulgidus Rio2 and crystal structures of its nucleotide-metal ion complexes. *FEBS J.* **272**, 2800–2810 (2005).
3. Ameismeier, M., Cheng, J., Berninghausen, O. & Beckmann, R. Visualizing late states of human 40S ribosomal subunit maturation. *Nature* **558**, 249–253 (2018).
4. Ben-Shem, A. *et al.* The structure of the eukaryotic ribosome at 3.0 Å resolution. *Science* **334**, 1524–1529 (2011).
5. Scaiola, A. *et al.* Structure of a eukaryotic cytoplasmic pre-40S ribosomal subunit. *EMBO J.* **37**, (2018).
6. Ye, Q., Ur, S. N., Su, T. Y. & Corbett, K. D. Structure of the Saccharomyces cerevisiae Hrr25:Mam1 monopolin subcomplex reveals a novel kinase regulator. *EMBO J.* **35**, 2139–2151 (2016).
7. Thomas, B. J. & Rothstein, R. Elevated recombination rates in transcriptionally active DNA. *Cell* **56**, 619–630 (1989).
8. Mitterer, V. *et al.* Sequential domain assembly of ribosomal protein S3 drives 40S subunit maturation. *Nat. Commun.* **7**, 10336 (2016).
9. Brachmann, C. B. *et al.* Designer deletion strains derived from Saccharomyces cerevisiae S288C: a useful set of strains and plasmids for PCR-mediated gene disruption and other applications. *Yeast Chichester Engl.* **14**, 115–132 (1998).
10. James, P., Halladay, J. & Craig, E. A. Genomic libraries and a host strain designed for highly efficient two-hybrid selection in yeast. *Genetics* **144**, 1425–1436 (1996).

11. Gietz, R. D. & Sugino, A. New yeast-Escherichia coli shuttle vectors constructed with in vitro mutagenized yeast genes lacking six-base pair restriction sites. *Gene* **74**, 527–534 (1988).

12. Schäfer, T., Strauss, D., Petfalski, E., Tollervey, D. & Hurt, E. The path from nucleolar 90S to cytoplasmic 40S pre-ribosomes. *EMBO J.* **22**, 1370–1380 (2003).

13. Ferreira-Cerca, S. *et al.* ATPase-dependent role of the atypical kinase Rio2 on the evolving pre-40S ribosomal subunit. *Nat. Struct. Mol. Biol.* **19**, 1316–1323 (2012).
