# Supplementary material for: Conformational proofreading of distant 40S ribosomal subunit maturation events by a long-range communication mechanism
Source: Nat Commun. 2019 Jun 21;10:2754. doi: 10.1038/s41467-019-10678-z (PMC6588571; doi:10.1038/s41467-019-10678-z)
Supplement: Supplementary file 3 — Reporting Summary [file 41467_2019_10678_MOESM3_ESM.pdf]

nature research

Corresponding author(s): Sebastien Ferreira-Cerca, Dieter Kressler, Celia Plisson-Chastang and Brigitte Pertschy

Last updated by author(s): Apr 29, 2019

Reporting Summary

Nature Research wishes to improve the reproducibility of the work that we publish. This form provides structure for consistency and transparency in reporting. For further information on Nature Research policies, see Authors & Referees and the Editorial Policy Checklist.

Statistics

For all statistical analyses, confirm that the following items are present in the figure legend, table legend, main text, or Methods section.

n/a	Confirmed	
☐	☒	The exact sample size (n) for each experimental group/condition, given as a discrete number and unit of measurement
☐	☒	A statement on whether measurements were taken from distinct samples or whether the same sample was measured repeatedly
☒	☐	The statistical test(s) used AND whether they are one- or two-sided<br>*Only common tests should be described solely by name; describe more complex techniques in the Methods section.*
☒	☐	A description of all covariates tested
☒	☐	A description of any assumptions or corrections, such as tests of normality and adjustment for multiple comparisons
☐	☒	A full description of the statistical parameters including central tendency (e.g. means) or other basic estimates (e.g. regression coefficient) AND variation (e.g. standard deviation) or associated estimates of uncertainty (e.g. confidence intervals)
☒	☐	For null hypothesis testing, the test statistic (e.g. F, t, r) with confidence intervals, effect sizes, degrees of freedom and P value noted<br>*Give P values as exact values whenever suitable.*
☒	☐	For Bayesian analysis, information on the choice of priors and Markov chain Monte Carlo settings
☒	☐	For hierarchical and complex designs, identification of the appropriate level for tests and full reporting of outcomes
☒	☐	Estimates of effect sizes (e.g. Cohen's d, Pearson's r), indicating how they were calculated

Our web collection on statistics for biologists contains articles on many of the points above.

Software and code

Policy information about availability of computer code

Data collection	cryo-EM images data acquisition was performed using the EPU (Thermo Fisher Scientific) software
Data analysis	cryo-EM single particle analysis was performed using Relion 2.1<br>Atomic model was derived from the cryo-EM maps using Coot, REFMAC5, Phenix RealSpaceRefine.

For manuscripts utilizing custom algorithms or software that are central to the research but not yet described in published literature, software must be made available to editors/reviewers. We strongly encourage code deposition in a community repository (e.g. GitHub). See the Nature Research guidelines for submitting code & software for further information.

Data

Policy information about availability of data

All manuscripts must include a data availability statement. This statement should provide the following information, where applicable:

- Accession codes, unique identifiers, or web links for publicly available datasets
- A list of figures that have associated raw data
- A description of any restrictions on data availability

Cryo-EM maps have been deposited in the Electron Microscopy Data Bank (EMDB), under the accession codes: EMD-4792 (C1-S20Δloop); EMD-4793 (C2-S20Δloop); EMD-4794 (C1-Head only); EMD-4795 (C2-Head only); EMD-4796 ("Dim1"). Two atomic coordinate models have been deposited in the PDB with accession codes PDB: 6RBD; PDB: 6RBE, corresponding to the C1-S20Δloop and C2-S20Δloop maps, respectively.

Field-specific reporting

Please select the one below that is the best fit for your research. If you are not sure, read the appropriate sections before making your selection.

☒ Life sciences ☐ Behavioural & social sciences ☐ Ecological, evolutionary & environmental sciences

For a reference copy of the document with all sections, see nature.com/documents/nr-reporting-summary-flat.pdf

Life sciences study design

All studies must disclose on these points even when the disclosure is negative.

Sample size	Sample size was adequate - all effects were clear and reproducible in all analyzed samples
Data exclusions	for the cryo-EM analyses, the number of particles excluded form the analysis is indicated in Supplementary Figure 7; no other data were excluded
Replication	qualitative results were reproduced in at least a second experiment; for quantitative results details on the number of replicates are given in the manuscript text; all replications of experiments were successfull
Randomization	no randomization was performed. Only yeast strains with defined genotypes were used as listed in Supplementary Table 2.
Blinding	no blinding was performed; data are not subjective and all outcomes are presented

Reporting for specific materials, systems and methods

We require information from authors about some types of materials, experimental systems and methods used in many studies. Here, indicate whether each material, system or method listed is relevant to your study. If you are not sure if a list item applies to your research, read the appropriate section before selecting a response.

Materials & experimental systems

n/a	Involved in the study
☐	☒ Antibodies
☒	☐ Eukaryotic cell lines
☒	☐ Palaeontology
☒	☐ Animals and other organisms
☒	☐ Human research participants
☒	☐ Clinical data

Methods

n/a	Involved in the study
☒	☐ ChIP-seq
☒	☐ Flow cytometry
☒	☐ MRI-based neuroimaging

Antibodies

Antibodies used	anti-Rps3 antibody (1:30,000; provided by Matthias Seedorf), anti-Nob1 antibody (1:5,000; provided by David Tollervey), anti-Ltv1 antibody (1:8,000; provided by Katrin Karbstein), anti-Enp1 antibody (1:4,000; provided by Katrin Karbstein), anti-Tsr1 antibody (1:4,000; provided by Katrin Karbstein), anti-Dim1 antibody (1:4,000; provided by Katrin Karbstein), anti-Pno1 antibody (1:2,000; provided by Katrin Karbstein), anti-Rio2-antibody (1:5,000; provided by Katrin Karbstein), anti-Hrr25 antibody (1:5,000; provided by Wolfgang Zachariae), anti-CBP antibody (1:5,000; Merck-Millipore, Cat.Nr. 07-482), secondary anti-rabbit horseradish peroxidase-conjugated antibody (1:15,000; Sigma-Aldrich, Cat.Nr. A7058), horseradish-peroxidase-conjugated anti-HA antibody (1:5,000; Roche, Cat.Nr. 12013819001).
Validation	most of the antibodies are gifts from other research group - no validation reports are available; three antibodies are commercially available, catalogue numbers are given above
