# Supplementary material for: Conformational proofreading of distant 40S ribosomal subunit maturation events by a long-range communication mechanism
Source: Nat Commun. 2019 Jun 21;10:2754. doi: 10.1038/s41467-019-10678-z (PMC6588571; doi:10.1038/s41467-019-10678-z)
Supplement: Supplementary file 2 — Peer Review File [file 41467_2019_10678_MOESM2_ESM.pdf]

Reviewers' comments:

Reviewer #1 (Remarks to the Author):

Mitterer et al. have dissected late stages of pre-40S maturation by studying the interplay between Ltv1, Rio2 and ribosomal proteins S3 and S20 that ultimately results in the phosphorylation of Ltv1 by Hrr25.

The combination of yeast genetics and biochemistry provide solid evidence for the presented model of how these factors interact during late pre-40S biogenesis. It is the opinion of this reviewer that these data are of general interest to the broad readership of Nature Communications that should be published provided that the following comments are addressed:

1. In the introduction the authors correctly refer to the earliest particles as 90S particles. As described in the original literature (and also reviewed in Rorbach & Amunts, NSMB 2017), these particles contain a 35S pre-ribosomal RNA. By contrast, more recent high-resolution cryo-EM structures (Barandun et al. & Cheng et al. 2017) have described the small subunit processome, a precursor of the small ribosomal subunit only. As there is now a proven absence of any 35S pre-rRNA in any of these structures, the term "small subunit processome" should be used for these particles whereas the term "90S" should be used precisely for what it originally describes, a precursor containing the 35S pre-rRNA.

2. A set of complementary data was recently published on human pre-40S particles (Ameismeier et al. 2018). The resulting structural snapshots (while from a different organism and without any mechanistic data) are largely in agreement with the proposed model of the authors of this manuscript and should be incorporated as complementary evidence earlier in the manuscript. More specifically, some of this data may be directly relevant to the current manuscript. For example, in Ameismeier et al. density for a protein is shown near Enp1/Ltv1, which is listed as "Factor X". Since SDS-PAGE analysis in the supplementary data indicates that two isoforms of caseine kinase (KC1D and KC1E) and PARN (a nuclease) are the only late factors present in the sample, it is possible that Factor X may be caseine kinase, the human homologue of Hrr25.

3. The authors have designed specific mutations of S20 (a loop deletion and other more specific mutations) and pre-40S particles were purified (via Tsr1) and later analyzed for the presence of many different assembly factors (Western blots Figures 3G, 6A-C). However, at no point is the incorporation of S20 and its mutants compared. Without a corresponding western blot, how can the authors rule out that S20 mutants are simply not properly incorporated into the pre-40S particles?
4. The authors should show a protein gel of the purified pre-40S particle containing the S20 loop deletion mutant in either Figure 1 or the supplement.

5. Similar to points 3 and 4, the presence of Hrr25 is not verified by western blots in any of the figures where all other components of purified pre-40S particles are analyzed. Especially in Figure 6A-C, the relative amounts of Hrr25 would be incredibly useful to see if the level of phosphorylation is simply correlated with Hrr25 recruitment or other factors.

6. The weakest data in this manuscript are the 3D reconstructions obtained by cryo-EM. Despite extensive data collection with up to 5000 images, the resulting resolution of the obtained reconstructions is surprisingly low with the highest resolution reported around 6.6 Angstroms. Given the level of detail that is required to observe and interpret the structural consequences of the introduced mutations (such as the mutations of the S20 loop), this data is not particularly useful. There are several points that need to be addressed:

a. Given the rather strict criterion to use only images with CTF fits of 5 Angstroms or better, have the authors tried to include more of the original particles (i.e. using a CTF cutoff of for example 8

Angstroms) such that the particle number could be increased?

b. Apart from FSC curves, there is no additional way to assess resolution in the supplement. The authors should use commonly used coloring schemes to indicate the resolutions of the particles including slabs through the centers of the reconstructions. In addition the distribution of views for each reconstruction should be shown.

c. All cryo-EM reconstructions (Figures 4 and 5) are contoured at a level where structural features of the body are lost while noise levels in the head region are clearly apparent. The supplementary data clearly indicates flexibility in the head region. With more particles the authors should try masked 3D classifications to obtain a more defined head region. The contour levels in Figures 4 and 5 should be adjusted such that normal features such as RNA helices etc. are clearly visible.

d. In Figure 5 panels B and C, the limited resolutions of these reconstructions do not permit a detailed mechanistic interpretation of the conformations of Rio2 beyond rigid body docking. Using molecular dynamics flexible fitting to postulate side chain orientations (Figure 5D) seems unreasonable and this data should not be used. Similarly modeling steric clashes by using a Phyre model does not seem appropriate.

e. On page 9, line 298 the authors mention that in Figure 4 panel D a segment is shown in violet. The coloring in the figure should be changed to improve its clarity.

f. On page 9, lines 299-301 the authors refer to Figure S6 panels E and F. These panels are currently not clear at all so that the argument that one fit is better than the other cannot be made. These panels should be improved for clarity.

Reviewer #2 (Remarks to the Author):

This study aims to unravel the mechanism leading to the release of Ltv1 and Rio2 from pre-40S particles in yeast. Through a combination of genetics, biochemical assays and cryo-EM the authors try to provide sufficient evidence that during pre-40S maturation contacts between Rps20 and Rps3 permits assembly factor Ltv1 to recruit the Hrr25 kinase for subsequent Ltv1 phosphorylation. As part of the mechanisms to mediate the release of Rio2 and Ltv1, a deeply buried Rps20 loop reaches to the opposite pre-40S side of the head, where it stimulates Rio2 ATPase activity. Both events combined induce releasing of Rio2 and phosphorylated Ltv1. The authors propose a model by which Rps20 senses the conformation around its binding site and also around the Rio2 binding site and ensures the release of Ltv1 and Rio2 is only triggered once all necessary maturations steps have been completed.

I found that in this study biochemical and genetic experiments support well the multiple conclusions of the study regarding the mechanisms of release of Ltv1 and Rio2. This will include the suggestion that binding of Hrr25 facilitates the release of Ltv1 or the important role played in the biogenesis of the pre-40S particles by the Rsp20 loop-region. The experiment showed in Fig 3G indicates that pre-40S particles purified from a rps20Δloop strain accumulate significantly higher amounts of Ltv1 and Rio2 strengthening the argument that the loop region of Rsp20 is directly involved in the release of the Rio2 factor.

A much weaker point of the study is the cryo-EM work, which in my view fails to provide strong structural validation of the findings obtained through genetics and biochemistry. To visualize the molecular mechanisms underlying the release defects for the assembly factors the authors obtained the cryo-EM structures of pre-40S particles purified through a Tsr1 bait from the rps20Δloop and wild type cells. Overall the obtained resolution for the structures is very limited (6-11Å). In addition of these resolutions not being considered state of the art today, more importantly they are certainly not

sufficient for the conclusions withdraw by the authors. Although the authors do not present any raw electron micrograph it seems the overall quality of the cryo-EM data collected may be limited. That is indicated by the fact that in the dataset collected for the WT pre-40S particles ~228,000 particles were picked up but only ~86,000 were left after the class2d analysis. A similar ratio was obtained for the rps20Δloop pre-40S particles. That would indicate that there are large number of contaminating/false positive particles in those electron micrographs. This is already impacting severely the quality of the final 3D reconstructions as cryo-EM maps are obtained from datasets of very limited size. The fact that for both samples multiple classes are found limits resolution even further, as now the limited number of particles gets even more split between the different classes.

In particular, the interpretation of the Rio2 density in the cryo-EM map obtained for the C3-S20Δloop class is one of the clear examples on the manuscript of over interpretation of the maps given the attained resolution. I don't agree the authors can conclude that in this structure Rio2 factor adopt an altered conformation. It is not a reliable practice to attempt flexible fitting in the Rio2 density at a resolution of almost 11Å for that map. Multiple equally likely solutions are possible at that resolution. To propose that Rio2 adopt an 'unwound' conformation in this structure is simply not supported by the quality of the data as no elements of secondary structure are visible at that resolutions. I also believe based on Supl. Fig 5 that that the cryo-EM map for C3-S20Δloop class represent a mixture of multiple classes and further classification may be required. That is indicated by the fact that even though the number of particles incorporated in this map is the highest (~39,000), the obtained resolution is the lowest (10.6Å). Other classes such as C1-wt containing only 7,000 particles reach 10.3Å resolution.

A few other concerns regarding the image processing approaches I would like the authors to comment on:

1. It is not clear why in step B of the classifications images with no binning are used. Classification algorithms rely in low frequencies to sort out the particles. Consequently, performing the classification with bin images typically accelerate the processing and at the same time classification performance is also typically better.

2. Seems that the cryo-EM structures presented in the figures have not undergone sharpening or postprocessing. At least this step is not detailed on the material and methods. This approach is appropriate with those structures above 10Å resolution, However, structures around 6Å would benefit and will gain more detail after some sharpening is performed.

Consequently, I believe require a significant improvement of the cryo-EM data and analysis before it can be accepted for publication in this journal.

Dear Reviewers,

thank you very much for your positive feedback on our manuscript. Your constructive suggestions helped us to significantly improve the quality of the manuscript. As you will see in the revised manuscript, we have performed all the suggested biochemical experiments. Moreover, we have completely re-done our cryo-EM investigations of Rps20Δloop pre-40S particles. We could gain access to a Titan Krios Microscope at the ESRF, which allowed us to obtain Rps20Δloop pre-40S cryo-EM structures with significantly improved resolution (3.5 and 3.8 Å). Comparison of these structures to previously published pre-40S particles revealed some interesting features that give new insights into pre-40S maturation (described in the revised manuscript). We hope you appreciate that, considering the main focus of our manuscript is on functional investigations, these structural analyses represented an immense effort and that an even more detailed investigation including additional particles (like wild-type Tsr1-TAP particles) would make up an independent story by itself.

In addition, we would like to mention that, although not requested by the reviewers, we have in the meantime more precisely mapped the Hrr25- and Enp1-interacting regions on Ltv1 by Y2H (i.e. we tested new Ltv1 fragments starting at amino-acid 160 for interaction with both Hrr25 and Enp1), and incorporated these new data into Figure 1e and Supplementary Figure 2.

A point-by-point response to your comments is given below.

Reviewer #1:

1. In the introduction the authors correctly refer to the earliest particles as 90S particles. As described in the original literature (and also reviewed in Rorbach & Amunts, NSMB 2017), these particles contain a 35S pre-ribosomal RNA. By contrast, more recent high-resolution cryo-EM structures (Barandun et al. & Cheng et al. 2017) have described the small subunit processome, a precursor of the small ribosomal subunit only. As there is now a proven absence of any 35S pre-rRNA in any of these structures, the term "small subunit processome" should be used for these particles whereas the term "90S" should be used precisely for what it originally describes, a precursor containing the 35S pre-rRNA.

<u>Response</u>: We are aware of this controversy of how to properly refer to these earliest pre-ribosomal particles. We have therefore, upon first mentioning these particles, now indicated both terms, SSU processome and 90S particles. Unfortunately, neither 90S particles nor the SSU processome have been clearly defined and the same names have been used to describe particles with different rRNA and protein compositions before. Since our study focuses on the functional dissection of a pre-40S maturation step, we do not consider it necessary to give further explanations concerning the differences between SSU processomes and 90S particles.

2. A set of complementary data was recently published on human pre-40S particles (Ameismeier et al. 2018). The resulting structural snapshots (while from a different organism and without any mechanistic data) are largely in agreement with the proposed model of the authors of this manuscript and should be incorporated as complementary evidence earlier in the manuscript. More specifically, some of this data may be directly relevant to the current manuscript. For example, in Ameismeier et al. density for a protein is shown near Enp1/Ltv1, which is listed as "Factor X". Since SDS-PAGE analysis in the supplementary data indicates

that two isoforms of caseine kinase (KC1D and KC1E) and PARN (a nuclease) are the only late factors present in the sample, it is possible that Factor X may be caseine kinase, the human homologue of Hrr25.

Response: We now discuss the Ameismeier *et al.* paper more extensively and also mention the paper already in the Introduction. Indeed, our new cryo-EM structures reveal a density in one pre-40S particle class from our *rps20*Δloop mutant that resembles Factor X with respect to its shape and positioning within the particle. The possibility that this factor corresponds to Hrr25 is now discussed both in the Results and the Discussion section.

3. The authors have designed specific mutations of S20 (a loop deletion and other more specific mutations) and pre-40S particles were purified (via Tsr1) and later analyzed for the presence of many different assembly factors (Western blots Figures 3G, 6A-C). However, at no point is the incorporation of S20 and its mutants compared. Without a corresponding western blot, how can the authors rule out that S20 mutants are simply not properly incorporated into the pre-40S particles?

Response: This is a good point. As we do not have an antibody against Rps20 (and the antibody against human S20 does not work to detect the yeast protein), we constructed strains with Rps20 variants fused to an N-terminal HA-tag (note that C-terminal fusions are not functional) to facilitate detection by Western blotting. These results confirm that all Rps20 variants are incorporated into pre-40S particles (shown in the new Supplementary Fig. 4).

4. The authors should show a protein gel of the purified pre-40S particle containing the S20 loop deletion mutant in either Figure 1 or the supplement.

Response: We do not want to show the gel in Figure 1 as the S20 loop deletion mutant has not been introduced yet at this point of the manuscript. However, we have included a Coomassie-stained gel of Tsr1-TAP particles purified from the *rps20*Δloop mutant in the new Supplementary Fig. 5.

5. Similar to points 3 and 4, the presence of Hrr25 is not verified by western blots in any of the figures where all other components of purified pre-40S particles are analyzed. Especially in Figure 6A-C, the relative amounts of Hrr25 would be incredibly useful to see if the level of phosphorylation is simply correlated with Hrr25 recruitment or other factors.

Response: We agree that this was a weak point of the submitted manuscript. The reason is that we did not have an Hrr25 antibody available and our attempts to generate fully functional tagged versions of Hrr25 failed. Thanks to the generous gift of Wolfgang Zachariae, we have in the meantime received an anti-Hrr25 antibody. We have now included anti-Hrr25 Western blots in the new Figure 4a-c (formerly 6a-c).

6. The weakest data in this manuscript are the 3D reconstructions obtained by cryo-EM. Despite extensive data collection with up to 5000 images, the resulting resolution of the obtained reconstructions is surprisingly low with the highest resolution reported around 6.6 Angstroms. Given the level of detail that is required to observe and interpret the structural consequences of the introduced mutations (such as the mutations of the S20 loop), this data is not particularly useful.

Response: Indeed, the resolution of the former cryo-EM structures was not very satisfying. We believe that the main reason was our limited access to high-end microscopes, and poor

choice of the camera used. Indeed, the images for the former *rps20*Δloop structures were taken with a 200 kV Talos Arctica equipped with a Falcon III direct electron detector operating in linear mode. In the meantime, we could however record new *rps20*Δloop images on the CM01 Titan Krios microscope at the ESRF (equipped with a K2 summit direct electron detector, and operating in counting mode), resulting in a much better resolution. The two different classes of Rps20Δloop particles have resolutions at an average of 3.5 and 3.8 Å, respectively. The body domain of these pre-40S particles is especially very well resolved (3.0 to 3.2 Å). The new structures revealed some interesting features of Rps20Δloop particles, which are described in the Results section of the revised manuscript. These two high resolution Rps20Δloop structures are now compared to already available near-atomic resolution structures of cytoplasmic pre-40S, but also to one another. Notably, Rps20Δloop particles are trapped in distinct maturation stages not observed before, and therefore give interesting new insights into the early cytoplasmic steps of pre-40S maturation. We are convinced that these insights add significant extra value to the manuscript.

a. Given the rather strict criterion to use only images with CTF fits of 5 Angstroms or better, have the authors tried to include more of the original particles (i.e. using a CTF cutoff of for example 8 Angstroms) such that the particle number could be increased?

Response: We had indeed tried to lower our threshold for CTF resolution cutoff and increase the number of particles in our reconstructions, but without improving final resolution of our 3D reconstructions. As stated above, we believe this lack of resolution was due to a bad strategy for image acquisition: in order to increase the number of micrographs (and thus the number of initial particles), we chose to acquire images on a direct electron detector operating in linear mode. In contrast, with the Titan Krios, which we used for the revision of this manuscript, the DED is operated in counting mode as a standard basis. This allowed us to record a dataset of micrographs, where only a few had their resolution limit worse than 4Å, or were of poor quality because of contaminants or overfocus (439/6480 recorded micrographs, as stated in the Material and Methods section and in Supplementary Fig. 7a). This allowed us to reach near-atomic resolution in our 3D reconstructions of *rps20*Δloop pre-40S particles.

b. Apart from FSC curves, there is no additional way to assess resolution in the supplement. The authors should use commonly used coloring schemes to indicate the resolutions of the particles including slabs through the centers of the reconstructions. In addition the distribution of views for each reconstruction should be shown.

Response: With our new dataset and 3D reconstructions, we are now showing a coloring scheme to indicate local resolution of the cryo-EM maps (Supplementary Fig. 7d). This local resolution assessment was performed using the ResMap software (Kucukelbir et al., 2014), as stated in the Material and Methods section.

c. All cryo-EM reconstructions (Figures 4 and 5) are contoured at a level where structural features of the body are lost while noise levels in the head region are clearly apparent. The supplementary data clearly indicates flexibility in the head region. With more particles the authors should try masked 3D classifications to obtain a more defined head region. The contour levels in Figures 4 and 5 should be adjusted such that normal features such as RNA helices etc. are clearly visible.

Response: So far, all our 3D reconstructions of cytoplasmic pre-40S particles (either at low or near-atomic resolution) hint at the higher flexibility/dynamism of the head compared to the body of the particles. This is particularly striking for the first “consensus” 3D structure of our

new dataset (Supplementary Figure 7a), where the body is resolved up to 3.1 Å resolution, but the head features are hardly distinguishable. Thus, as suggested by Referee 1, we have tried masked classifications focused on the head, with or without signal subtraction for the body region. Focused 3D classifications with signal subtraction gave the most resolved features for the head, yielding the two structural classes C1-Head only and C2-Head only presented in Supplementary Fig. 7a, which were further auto-refined and post-processed to ~3.8 Å resolution according to RELION gold standard FSC. To get "full" 3D reconstructions of these two 3D classes, we then retrieved the particles from each class in the dataset without signal subtraction, and auto-refined them separately; this yielded the C1- and C2-S20Δloop maps and models (Supplementary Fig. 7a, left panels), solved to overall resolutions of 3.5 and 3.8 Å, respectively. In these full 3D structures, the resolution of the head is slightly lower than in the "head only" reconstructions, which suggests that the head has a non-negligible swiveling movement compared to the body. This image analysis strategy is also described in the Material and Methods section.

d. In Figure 5 panels B and C, the limited resolutions of these reconstructions do not permit a detailed mechanistic interpretation of the conformations of Rio2 beyond rigid body docking. Using molecular dynamics flexible fitting to postulate side chain orientations (Figure 5D) seems unreasonable and this data should not be used. Similarly modeling steric clashes by using a Phyre model does not seem appropriate.

Response: We agree that these were rather speculative models. We removed them. In the new Rps20Δloop structures, Rio2 is well resolved in one of the classes (C1), allowing unbiased interpretation of the orientation. In contrast, Rio2 is very flexible in the second class (C2), preventing to make a reasonable model.

e. On page 9, line 298 the authors mention that in Figure 4 panel D a segment is shown in violet. The coloring in the figure should be changed to improve its clarity.

f. On page 9, lines 299-301 the authors refer to Figure S6 panels E and F. These panels are currently not clear at all so that the argument that one fit is better than the other cannot be made. These panels should be improved for clarity.

Response: As we have completely re-made the cryo-EM Figures for the submission of the revised manuscript, these points do not apply anymore.

Reviewer #2:

A much weaker point of the study is the cryo-EM work, which in my view fails to provide strong structural validation of the findings obtained through genetics and biochemistry. To visualize the molecular mechanisms underlying the release defects for the assembly factors the authors obtained the cryo-EM structures of pre-40S particles purified through a Tsr1 bait from the rps20Δloop and wild type cells. Overall the obtained resolution for the structures is very limited (6-11Å). In addition of these resolutions not being considered state of the art today, more importantly they are certainly not sufficient for the conclusions withdraw by the authors. Although the authors do not present any raw electron micrograph it seems the overall quality of the cryo-EM data collected may be limited. That is indicated by the fact that in the dataset collected for the WT pre-40S particles ~228,000 particles were picked up but only ~86,000 were left after the class2d analysis. A similar ratio was obtained for the rps20Δloop pre-40S particles. That would indicate that there are large number of

contaminating/false positive particles in those electron micrographs. This is already impacting severely the quality of the final 3D reconstructions as cryo-EM maps are obtained from datasets of very limited size. The fact that for both samples multiple classes are found limits resolution even further, as now the limited number of particles gets even more split between the different classes.

In particular, the interpretation of the Rio2 density in the cryo-EM map obtained for the C3-S20Δloop class is one of the clear examples on the manuscript of over interpretation of the maps given the attained resolution. I don't agree the authors can conclude that in this structure Rio2 factor adopt an altered conformation. It is not a reliable practice to attempt flexible fitting in the Rio2 density at a resolution of almost 11Å for that map. Multiple equally likely solutions are possible at that resolution. To propose that Rio2 adopt an 'unwound' conformation in this structure is simply not supported by the quality of the data as no elements of secondary structure are visible at that resolutions. I also believe based on Supl. Fig 5 that that the cryo-EM map for C3-S20Δloop class represent a mixture of multiple classes and further classification may be required. That is indicated by the fact that even though the number of particles incorporated in this map is the highest (~39,000), the obtained resolution is the lowest (10.6Å). Other classes such as C1-wt containing only 7,000 particles reach 10.3Å resolution.

<u>Response</u>: We agree that the cryo-EM was the weakest point of the study. In the meantime, we have greatly improved the resolution of the Rps20Δloop structures. We again find two main classes of particles that resemble the classes we described before, but due to the improved resolution we are now able to really draw conclusions about the structures. Please see also the response to reviewer 1.

1. It is not clear why in step B of the classifications images with no binning are used. Classification algorithms rely in low frequencies to sort out the particles. Consequently, performing the classification with bin images typically accelerate the processing and at the same time classification performance is also typically better.

<u>Response</u>: In the first version of the paper, we had lower quality datasets, and the processing scheme we used was the fruit of many trial-and-error assays, aiming at having the best possible 3D classes and subsequently highest resolution auto-refined 3D maps. In that case, we found that the most stable 3D classes, with the highest final resolution were obtained with a binning of 1 (and a search angle of 3.5°). However, with our new dataset, initial 2D and 3D classifications were performed with a binning of 8 and 4, respectively (as stated in the Material and Methods section), and this strategy led us to isolate two pools of particles, which yielded final 3D maps solved to 3.5 and 3.8 Å resolution.

2. Seems that the cryo-EM structures presented in the figures have not undergone sharpening or postprocessing. At least this step is not detailed on the material and methods. This approach is appropriate with those structures above 10Å resolution, However, structures around 6Å would benefit and will gain more detail after some sharpening is performed.
Consequently, I believe require a significant improvement of the cryo-EM data and analysis before it can be accepted for publication in this journal.

<u>Response</u>: Indeed, we did not sharpen the maps under 10 Å resolution in the first version of the manuscript, because we could not find the right balance between more details in the body region vs. more noise in the head region. We also showed unsharpened maps so that all the cryo-EM maps, solved between 11 and 6 Å resolution, looked coherent with each

other. We agree that this was probably a poor choice. With our new dataset however we have been able to reach near-atomic resolutions, and post-processed the maps either in RELION for reconstructions of the head and Dim1 region with signal subtraction, but also, for full reconstructions (C1- and C2-S20Δloop) with the LocScale software developed by Arjen Jakobi (Jakobi et al., 2016). Local B-factors are calculated from an initial atomic model, and subsequently imposed on maps for their sharpening. Indeed, full reconstructions (see Supplementary Fig. 7a), showed less well resolved features for the head compared to the body, so in this case imposing a single B-factor to the whole map would lead either to under-sharpening of the body, or over-sharpening of the head. This process is also mentioned in the Material and Methods section.

REVIEWERS' COMMENTS:

Reviewer #1 (Remarks to the Author):

The authors have significantly improved this manuscript and completely redone all the cryo-EM work, which has now allowed them to use maps at near-atomic resolution for their analyses. All my previous concerns have been addressed and in my opinion this manuscript is now ready for publication in Nature Communications once the last following items are addressed:

1. In Figure 5, the authors now observe density near rpS3 and rpS20, which was previously observed in human pre-40S particles without identification (Ameismeier et al.) Since the authors have clearly shown that Hrr25 is the only missing factor for which no density has been assigned, and since they fitted a model for Hrr25 in their maps and discuss this in the text, I believe it would be appropriate to label this density as "(Hrr25)" in the figure so that this part is clarified for the scientific community.

2. The modeling of the crystal structure of rps3 in figures 6c and supplementary figure 10a could be improved to better fit the cryo-EM maps. Since the crystal structure of rpS3 in complex with Yar1 (PDB 4BSZ) contains a swapped beta-sheet within the C-terminal domain, the fitted C-terminal domain in figures 6c and supplementary figure 10a lacks these elements of the structure and this could be fixed by simply fitting an intact C-terminal domain of rpS3 (either with the fixed coordinates of 4BSZ or with the repositioned rpS3 domain from a 40S structure). Since these structures will serve as references in the future, I believe this would be very helpful.

3. For the cryo-EM processing, the distribution of views for each reconstruction should be shown.

Reviewer #2 (Remarks to the Author):

I appreciate the authors did not take any shortcut in addressing my concerns about the cryo-EM data. In the previous version of the manuscript the presented cryo-EM maps were at low resolution. The main reason was that the dataset collected to produce these data was of marginal quality due to a poor strategy during data collection.

I think it was good judgement from these authors to go back to the microscope and collect high quality data in a high-end system, instead of trying to extract non existing high-resolution information from images where it was not present in the first place.

The new cryo-EM reconstructions at much higher resolution certainly support now the claims made in the paper. Therefore, I believe now this manuscript should be accepted for publication.

Response to Referees

Reviewer #1 (Remarks to the Author):

The authors have significantly improved this manuscript and completely redone all the cryo-EM work, which has now allowed them to use maps at near-atomic resolution for their analyses. All my previous concerns have been addressed and in my opinion this manuscript is now ready for publication in Nature Communications once the last following items are addressed:

1. In Figure 5, the authors now observe density near rpS3 and rpS20, which was previously observed in human pre-40S particles without identification (Ameismeier et al.) Since the authors have clearly shown that Hrr25 is the only missing factor for which no density has been assigned, and since they fitted a model for Hrr25 in their maps and discuss this in the text, I believe it would be appropriate to label this density as "(Hrr25)" in the figure so that this part is clarified for the scientific community.

Response: We appreciate the support of our interpretation of these data. As however, Hrr25 is only a likely guess but cannot be assigned unambiguously, we prefer to keep the label as factor X and to discuss in the text why Hrr25 is, based on current knowledge, the most likely candidate.

2. The modeling of the crystal structure of rps3 in figures 6c and supplementary figure 10a could be improved to better fit the cryo-EM maps. Since the crystal structure of rpS3 in complex with Yar1 (PDB 4BSZ) contains a swapped beta-sheet within the C-terminal domain, the fitted C-terminal domain in figures 6c and supplementary figure 10a lacks these elements of the structure and this could be fixed by simply fitting an intact C-terminal domain of rpS3 (either with the fixed coordinates of 4BSZ or with the repositioned rpS3 domain from a 40S structure). Since these structures will serve as references in the future, I believe this would be very helpful.

Response: Thank you for this good suggestion. Indeed, we overlooked this slight incorrectness. We now exchanged the C-terminal part of Rps3 by fitting the C-terminal domain of mature Rps3, resulting indeed in an even better fit into the cryo-EM density. The Figures were adapted accordingly.

3. For the cryo-EM processing, the distribution of views for each reconstruction should be shown.

Response: We now included this information in Supplementary Figure 7.

Reviewer #2 (Remarks to the Author):

I appreciate the authors did not take any shortcut in addressing my concerns about the cryo-EM data. In the previous version of the manuscript the presented cryo-EM maps were at low resolution. The main reason was that the dataset collected to produce these data was of marginal quality due to a poor strategy during data collection.

I think it was good judgement from these authors to go back to the microscope and collect high quality data in a high-end system, instead of trying to extract non existing high-resolution information from images where it was not present in the first place.

The new cryo-EM reconstructions at much higher resolution certainly support now the claims made in the paper. Therefore, I believe now this manuscript should be accepted for publication.

Response: Thank you for this positive feedback.
